# Effects of dosing non-toxigenic Clostridia on the bacterial populations and immunological responses in the intestinal tract of lactating dairy cows

**DOI:** 10.3389/fmicb.2023.1107964

**Published:** 2023-06-15

**Authors:** Hye Won Kim, Na Kyung Kim, Jesse Thompson, Mackenzie de Jesus, Josh Rehberger, Thomas Rehberger, Alexandra Helena Smith, Roderick Ian Mackie

**Affiliations:** ^1^Department of Animal Sciences, University of Illinois at Urbana-Champaign, Urbana, IL, United States; ^2^Arm & Hammer Animal and Food Production, Waukesha, WI, United States; ^3^Carle R. Woese Institute for Genomic Biology, University of Illinois at Urbana-Champaign, Urbana, IL, United States

**Keywords:** Clostridia, lactating cows, dosing, intestinal tract, immune function

## Abstract

Understanding the effects of dosing non-toxigenic Clostridia to cows is rare and has received little attention so far. In the present study, a total of eight lactating dairy cows were divided in two groups: control (*n* = 4) or Clostridia challenged (oral supplementation of five diverse strains of *Paraclostridium bifermentans*, *n* = 4). Bacterial communities were analyzed by qPCR and next-generation sequencing (NGS) in the buccal mucosa as well as digesta and mucosal samples of the gastrointestinal (GI) tract from rumen to rectum (10 compartments), as well as fecal samples. Transcriptomic analysis of barrier and immune-related gene expression was performed on rumen, jejunum, and liver samples. We observed increased microbial populations with the Clostridial challenge in the buccal tissues and the proximal GI tract (forestomach), correlating with Clostridial loads in the feed. Otherwise, there were no significant differences in microbial populations (*p* > 0.05) throughout the distal part of the GI tract. The NGS approach, however, revealed that the Clostridial challenge changed the relative abundance of gut and fecal microbiota. In particular, in the challenge group, no *Bifidobacterium* was observed in the mucosa-associated microbiota and abundance of Pseudomonadota increased in the feces. These results indicated potential adverse effects of Clostridia to cow health. In general, immune responses to the Clostridial challenge were weak. However, transcriptional analysis revealed the down-regulation of junction adhesion molecule encoding gene (−1.44 of log_2_ fold-change), which might impact intestinal permeability.

## 1. Introduction

Clostridia are obligate anaerobic, gram-positive, spore-forming bacteria in the phylum Firmicutes (recently renamed as Bacillota). It is ubiquitous in diverse environments including soil, sewage, and the intestinal tract of both human and animal (Boulianne et al., 2020). This ubiquitous nature of clostridia especially in the farm environment means that cows can be exposed to the bacteria through dietary components. When dairy cows are fed with contaminated feed included in total mixed rations (TMR), the spore-forming Clostridia are concentrated in the gastrointestinal (GI) tract through the digestive processes and then pass to the feces (Vissers et al., 2007; Borreani et al., 2019). In a sequence based approach targeting 16S rRNA in cattle feces, Dowd et al. (2008) detected 37 separate species of Clostridia and approximately 20% of the total microbial populations were *Clostridium* spp. (Dowd et al., 2008). Another study also reported the Clostridiales (order) in digesta and mucosa across 10 sites in the intestinal tract of cows, although there was a low relative abundance of this taxa (Mao et al., 2015).

Under certain conditions and predisposing host factors, certain species of Clostridia can produce toxins causing disease in cattle. One of the most prominent pathogens causing enteric disease is *Clostridium perfringens*, which is associated with necrotic enteritis, abomasal disease, and hemorrhagic bowel syndrome in cows (Simpson et al., 2018). The occurrence of *Clostridium* spp. in the farm environment and host animals, however, is not always a concern for dairy cows. Commensal Clostridial species are widely distributed in the gut of cattle and have a wide range of biochemical traits that could impact host health either positively or negatively. For example, *Paraclostridium bifermentans, C. beijerinckii,* and *C. butyricum* are non-toxigenic Clostridia found in farm soil, feed ingredient, intestinal tract of dairy cows, and feces (Borreani et al., 2019). Some of these *Clostridium* spp. may be beneficial and can be used as probiotics for cows, which would depend on the specific *Clostridium* species involved and their populations and abundance in the different compartments of GI tract.

The relationships between the bacterial communities in the GI tract and their host animals has provided deep insight into the benefits or risk of gut microbiota to the hosts (Mao et al., 2015) and previous studies suggested that assessing microbiota in various sections along the GI tract is essential (Malmuthuge et al., 2014; Mao et al., 2015). The complexity of digesta microbial communities in steers and the composition of digesta and mucosa-associated microbiota in the GI tract of calves have been reported (Malmuthuge et al., 2014). More recently, Mao et al. (2015) studied the bacterial communities in the GI tract of Holstein dairy cattle (Mao et al., 2015). These studies provided an additional understanding of the composition of GI tract microbiota in the animals; however, a systematic cultivation based approach together with the application of molecular ecology tools to identify the effects of certain bacterial species to the host has rarely been carried out with this compartment analysis. Thus, the objective of this study was to determine the effects of non-toxigenic Clostridia oral supplementation on gut microbiota of Holstein cows using a multiphasic approach.

Research on GI tract microbiota has been limited in the past due to their complexity and inability to culture obligate anaerobes (Maier et al., 2014). Improved sequencing and analytical techniques, however, now allow better resolution of microbial communities of the samples within a short time (Levy and Myers, 2016). To date, next-generation sequencing (NGS) platforms have mostly been used to specify the complex aspects of gastrointestinal microbiota in animals (Shang et al., 2018; Wirth et al., 2018; Wang et al., 2021), but this approach still has a limitation that the data can be shown as a relative abundance and used to calculate diversity indices. Microbiome analysis, therefore, should be performed with the exact quantification methods as well as viable cell counting and quantitative PCR approaches.

The present study was designed based on the hypothesis that naturally occurring Clostridia ingested by cows from the environment through diet can cause effects on the microbial populations along the GI tract and immunological responses of dairy cows. Our multiphasic research approach utilized (1) systematic cultivation of commensal Clostridia after oral supplementation with *P. bifermentans* and (2) analysis of microbiota and immune protein related genes by sample types (digesta vs. mucosa) and region-dependent bacterial segregation (compartment analysis of GI tract) (3) by utilizing viable cell counts, high-throughput real-time qPCR, and NGS.

## 2. Materials and methods

### 2.1. Ethical approval

The experimental design and animal use procedures were approved by the University of Illinois (Urbana-Champaign) Institutional Animal Care and Use Committee (no. 19036).

### 2.2. Animal handling and sample collection

Based on the randomized completed block design with 10 blocks per treatment (untreated control or commensal Clostridia treatment, *n* = 10 per treatment), a total of 20 mid-lactation [past peak, ~90 days in milk] Holstein cows were used in this animal study. Cows in each block were balanced with regard to parity, body condition score, and previous lactation milk yield to ensure these variables had a minimal chance of influencing the result. These cows did not have diarrhea and did not receive probiotics and antibiotics for the duration of this experiment. The first treatment served as the untreated control (no added Clostridia) and the second received a bolus of *P. bifermentans* (five diverse strains). The Clostridia challenge consisted of half of the daily dose administered orally by drench and the other half top-dressed on the TMR (7,179 ± 5,151 CFU/g of TMR) for 70 days. The ingredient composition of the lactation diet during the experimental period was as follows: corn silage (40.2% of dry matter), dry ground corn grain (17.3%), canola meal expelled (5.6%), alfalfa hay (18.4%), corn gluten feed pellets (7.9%), vitamin and mineral mix (4.4%), blood meal (0.4%), rumen-protected methionine (0.1%), urea 281 CP (0.4%), rumen inert fat (1.5%), and molasses (3.4%). During the challenge, two cows from the control group were excluded from the final dataset due to toxic mastitis and locomotion problems. A total of eight cows (four in each group) were randomly selected and then euthanized for tissue and microbiology sampling and the data presented in this paper. Sample information of the selected cows is presented in Supplementary Table S1. Specifically, mucosal and digesta samples were taken along the gastrointestinal tract at 10 sites of locations (rumen, reticulum, omasum, abomasum, duodenum, jejunum, ileum, cecum, colon, and rectum) at slaughter. Feed, fecal samples, and buccal tissue were also collected to analyze the initial microbiome in the samples, and liver tissue was collected to analyze immunological responses.

### 2.3. Viable counts of Clostridia

Clostridia were enumerated from tissue samples on tryptose sulfite cycloserine (TSC) agar (Oxoid, CM0587, Hampshire, UK) containing D-cycloserine (400 mg/L). Fecal samples were incubated at 60°C for 35 min and feed samples 50°C for 10 min prior to enumeration as described with the tissue samples. Agar plates were incubated under anaerobic conditions at 37°C for 24 h.

### 2.4. DNA extraction for qPCR

Each of the mucosal and digesta samples collected from the GI tract of cows was placed in a plastic centrifuge tube before experiments. Tissue samples (mucosa and liver) were cut (20 mg) and placed in the 1.5 mL EP tube, and then used to extract DNA by the DNeasy Blood & Tissue kit (Qiagen Inc., CA, United States) according to the manufacturer’s instructions. Otherwise, buccal DNA was extracted by using the Gentra Puregene Buccal Cell kit (Qiagen Inc.), which is designed to extract DNA from the buccal tissue. Total 200 mg of each digesta samples were used to extract DNA by using the QiAamp DNA Stool Min kit (Qiagen Inc.). For the nine feed samples, DNA was extracted using a DNeasy maricon Food Kit (Qiagen Inc.). All samples were duplicated, resulting in a total of 345 DNA samples to conduct the quantitative PCR in this study.

### 2.5. Quantitative PCR

#### 2.5.1. Empirical testing of the primers

The target bacterial taxa for the quantitative PCR were total bacteria, phylum Bacillota, phylum Bacteroidota, Clostridia, as well as *P. bifermentans, C. butyrium, C. beijerinkii*, and *C. perfringens* species. Primer sequences and reference bacteria used are presented in the Supplementary Table S2 (primer sequence used) and Supplementary Table S3 (reference bacteria used). Standard curves were established to quantify the target microorganisms. Briefly, reference cells grown on the nutrient medium or gut microbiota medium (GMM) at 37°C for 1–2 days were used to extract DNA. DNA was then serially diluted four-fold to generate a standard curve by qPCR. The regression equations of all the standard curves were presented in Supplementary Table S4 for which *R*^2^ ranged from 0.92 to 1.00.

#### 2.5.2. Fluidigm real-time qPCR

Each 384 well contained 20 μL reaction mixture with 10 μL of iQTM SYBR Green supermix, 300 nM of forward and reverse primers, 1 μL of DNA template, and 8 μL of pure H_2_O. The cycle conditions used in the fluidigm real-time qPCR (Fluidigm Corporation, CA, United States) were as follows: 95°C for 5 min, 40 cycles of 95°C for 15 s and 60°C for 1 min. Data from this experiment were collected through the real-time qPCR analysis software (QuantStudio™ Real-Time PCR Software ver. 1.3, Thermo Fisher Scientific, MA, USA). The Ct values obtained from the qPCR were calculated to the log copy number per gram of samples based on the standard curve established.

### 2.6. DNA extraction, library preparation, and sequencing

Extraction of genomic DNA was performed using the DNeasy PowerSoil HTP Kit (QIAGEN, Germantown, MD) with bead beating taking place for 2 min in a Mini-Beadbeater-96 (BioSpec Products, Bartlesville, OK). Genomic DNA was shipped to the Functional Genomics Unit at the Roy J. Carver Biotechnology Center for library preparation. PCR was performed for 35 cycles with 16S rRNA V4 primers for total bacteria (Walters et al., 2016) and SJ Clostridial specific primers (Hu et al., 2014) using the 48 Access Array IFC (Fluidigm, San Francisco, CA). The final size-selected amplicon pools were submitted to the DNA Services laboratory at the DNA Services laboratory at the Roy J. Carver Center at the University of Illinois at Urbana-Champaign. The final pools were quantified using Qubit (Life Technologies, Grand Island, NY) and then further quantified by qPCR on a BioRad CFX Connect Real-Time System (Bio-Rad Laboratories, Inc. CA), then pooled evenly. The pool was loaded onto 1 lane of a 2-lane HiSeq Rapid V2 flowcell at a concentration of 10 pM for cluster formation on the cBOT and then sequenced on an Illumina HiSeq 2,500 with version 2 Rapid SBS sequencing reagents. The libraries were sequenced from both ends of the molecules to a total read length of 250 nt from each end.

### 2.7. Sequence data processing

Sequence analysis was performed using QIIME2 version 2023.2 (Bolyen et al., 2019). Paired end sequences were merged, denoised, dereplicated and filtered of chimeras using DADA2 (Callahan et al., 2016). Taxonomy was assigned using a Naïve Bayes classifier trained to the EZBioCloud reference database, a highly curated database containing 16S sequences from both reference strains and derived from whole-genome assemblies (Kim et al., 2012). Sequences were trimmed to either the V4 or SJ primer sets, and the classify-sklearn (Pedregosa et al., 2011) method of the feature-classifier plugin (Bokulich, et al., 2018). Contamination from *Pseudomonas*, *Acinetobacter* and *Stenotrophomonas* was detected in negative control samples, so those genera were removed from the V4 results. Eukarya, Archaea and Bacteria with no taxonomic assignment at the phylum level were also removed from the V4 data. The SJ Clostridial specific primers were filtered to include only the class Clostridia.

Due to filtering to remove contamination, sequencing depth was lower than anticipated and lower abundance samples, especially those from the small intestines, were greatly impacted. To retain as many samples as possible alpha rarefaction plots of observed features were generated with a maximum sequencing depth of 4,000 reads. It was determined that the lowest possible number of reads required for retention in analysis while still covering diversity was 1,100 for the V4 primer data and 2000 for the Clostridia specific primers. Even with lowering the retention threshold, it was necessary to remove the duodenal samples from the analysis. Alpha diversity as determined by Shannon Entropy was compared between groups. There were no significant differences in alpha diversity between Control and High groups as determined by Kruskal-Wallis test. All alpha diversity measurements were measured at ASV level after filtering. Beta diversity was examined using Canoco5 (Microcomputer Power, Ithaca, NY) a multivariate statistical analysis program used in the ecology field (Šmilauer and Lepš, 2014). Count tables were log transformed and centered by species and Redundancy analysis (RDA), a linear constrained ordination method, was performed. Constrained ordination is a statistical method to relate multiple variables (e.g., species) to explanatory variables (e.g., gut section). RDA is used to visually represent the differences among samples, but in addition shows the fitted values of the species to explanatory variables similar to principal component analysis (PCA). Data are shown as biplots with the top best-fit species for a single explanatory variable. The explanatory variables are shown as a point, which would be the midpoint of the samples in that group while arrows represent species, with abundance increasing in the direction of the arrow. The amount of variation for the model was calculated as well as for each axis and the amount of variation attributable to each explanatory variable included in the model.

### 2.8. RT-PCR for immune-related genes

#### 2.8.1. cDNA preparation

The RNA was extracted from the rumen, jejunum, and liver tissue samples by using a miRNeasy Mini Kit (Qiagen Inc.) according to the manufacturer’s instruction. The yield of the total RNA was then measured using a Qubit (Invitrogen, Carlsbad, CA, United States). RNA was reverse-transcribed by High-Capacity cDNA Reverse Transcription Kits (Applied Biosystems, CA, United States) according to the manufacturer’s guide. cDNA was synthesized in a Thermo-cycler (Thermo Fisher Scientific) with 25°C for 10 min, 37°C for 120 min, and 85°C for 5 min, followed by a 4°C hold to stop the reaction.

#### 2.8.2. RT-PCR

Expresstion of the barrier function and inflammatory response related genes was evaluated by the fluidigm real-time PCR device as described above. The sequences of the primers used for the RT-PCR are listed in Supplementary Table S5. Relative gene expression levels of the Clostridial challenge group vs. control group were calculated by the comparative criticial threshold (2^−ΔΔCT^) method (Schmittgen and Livak, 2008). CMTM6, ERC1, and MRPL39 were used as housekeeping genes to normalize the input amounts of RNA and to determine the level of target gene expression (McCann et al., 2016).

### 2.9. Volatile fatty acids measurement

Rumen fluid samples were analyzed for volatile fatty acids by diluting rumen fluid 1:1 in 5 mM H_2_SO_4._ The mixture was homogenized then centrifuged at 22,000 g for 7 min. The supernatant was then put through two 0.22 μm filters. The VFA concentration in rumen fluid of cows was measured using a high performance liquid chromatograph (Shimadzu, Kyoto, Japan) equipped with a degasser, a LC-20AT pump, a SIL-20A autosampler, a CTO-20A column oven and an SPD-M20A diode array detector. The mobile phase used was 5 mM sulfuric acid and VFAs were separated on a Rezex ROA-Organic Acid H+ (8%) column (300 × 7.8 mm) from Phenomenex. The conditions for liquid chromatography were as follows: samples were eluted into 5 mM sulfuric acid and 1.0 μL of sample was injected into the oven at 67°C at a rate of 0.850 mL/min. The absorption spectra of the compounds was recorded between 190 and 500 nm. Data was processed using LabSolutions CS software (Shimadzu).

### 2.10. Statistical analysis

Plots were generated by R-Studio ver. 1.4.1717, SigmaPlot ver. 12.5, and excel 2016. Statistical analysis was performed using a SAS software version 9.4. Data were evaluated by a general linear model for variance analysis and Tukey’s proc. Hoc test was used to determine the significance of the differences among samples (^*^p < 0.05, ^**^p < 0.01, ^***^p < 0.001). The significance of each explanatory variable in the RDA model was determined by Bonferroni corrected *p*-value.

## 3. Results and discussion

**Figure 1:**
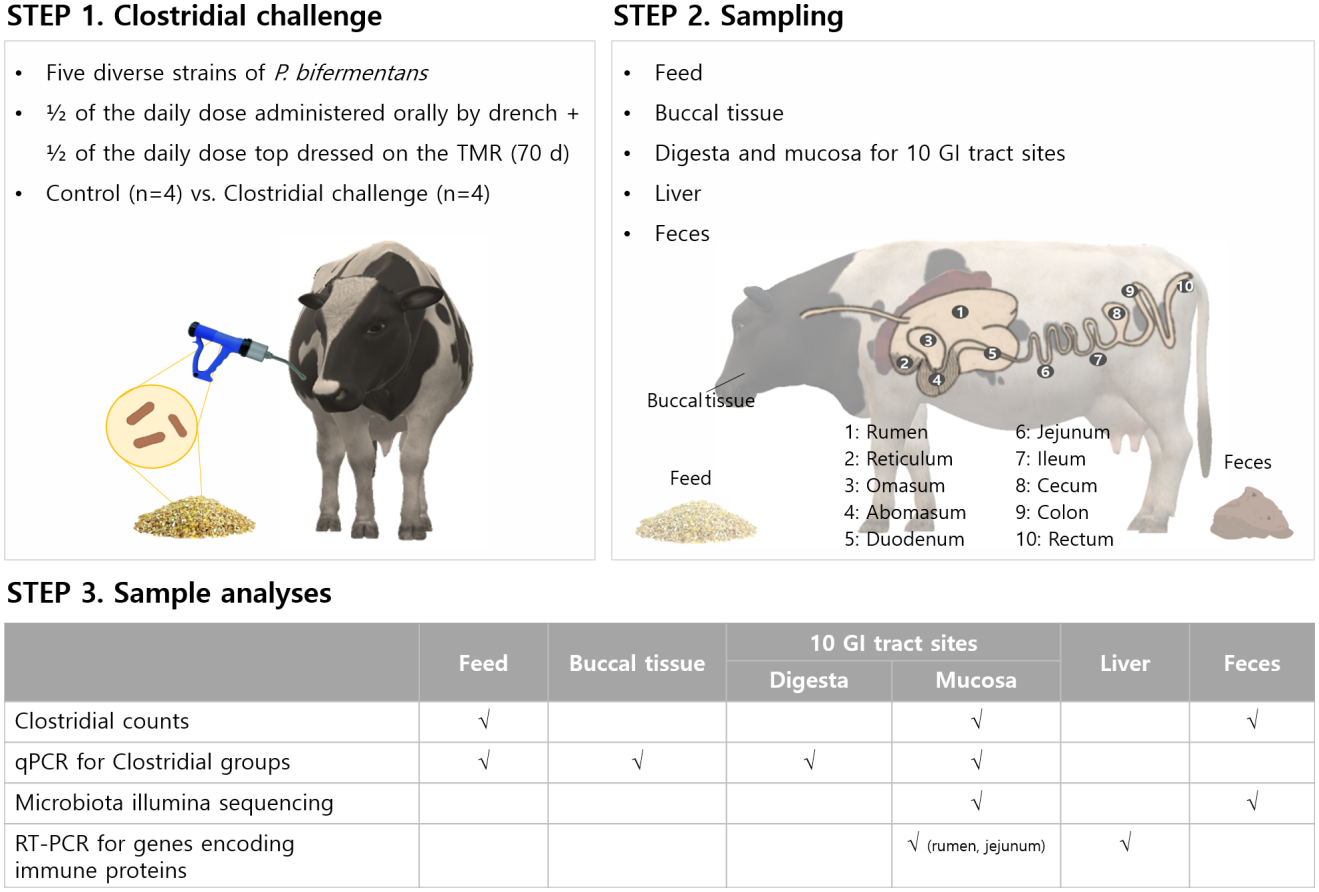
Diagram to illustrate the Clostridial challenge for cows and sampling points taken for microbial analyses, microbiome, and immune-response related gene expression.

This study characterized the composition and distribution of microbiota in digesta and mucosa in the GI tracts of Holstein dairy cows challenged with non-toxigenic Clostridia. Clostridial challenges to cows and all sampling points are shown in Figure 1. For the Clostridial challenge, five diverse strains of commensal *P. bifermentans* selected from 2,900 strains in the Arm and Hammer culture collection were used during the harvest.

### 3.1. Clostridial challenge

As previously described, Clostridia are ubiquitous and commonly found in the farm environment as well as in the diet fed to cows. According to a previous study regarding the distribution of the dominant species for the TMR contamination, Clostridia represented 52 to 63% of the total anaerobic sporeformers and the most frequently detected dominant species was *C. sporogenes* (Borreani et al., 2019). Another study reported that *C. perfringens* was the most dominant (64%) in the TMR followed by *P. bifermentans* (13%), *C. beijerinckii* (10%), and *C. butyricum* (5%). A preliminary internal study found that *P. bifermentans* (32.2%) was dominant followed by unidentified (30.0%), *C. beijerinckii* group (20.0%), *C. butyricum* (15.6%), and *C. perfringens* (2.2%) in TMR during the harvest of cows (Arm and Hammer, unpublished report). These results indicate that the Clostridial community in TMR differs by farm and might be affected by various other factors such as contaminated silage as well as soil contamination and supporting the concept of “microbial terroir” for between farm variation. In the present study, we tried to determine baseline Clostridial levels in the feed and their dose with commensal Clostridia to elevate these numbers and determine bacterial and immunological outcomes. Supplementary Figure S1A shows viable Clostridial counts in feed measured using a conventional plate counting apprach. Control feed was not analyzed for week 2 as samples were lost in transit. This was followed by a dramatic increase in week 3. However, after that, the levels were maintained at *ca.* 2 log CFU/g of the feed, while the Clostridial challenge group had 4 log CFU/g in the feed. Importantly, Clostridia in feed was consistently higher in the challenge group than in the control group by *ca.* 2 log CFU/g feed. The total clostridial challenge by feed was then calculated according as follows: Total daily Clostridial challenge (log CFU) = average daily intake (DMI, g) × Clostridia counts in feed (CFU/g). This calculation showed that the Clostridial challenge groups were supplemented with *ca.* 2 log more Clostridia than control groups which converts into *ca.* 100-fold higher bacterial concentration (Supplementary Figure S1B).

### 3.2. Compartment analysis of GI tract

Since the gastrointestinal tract-associated microbiota play an important biological role due to their close proximity to the host (Collado and Sanz, 2007), it is essential to understand their distribution throughout the GI tract. The compartmental analysis of the GI tract of cows has previously been investigated by culture-based methods or DNA techniques (Malmuthuge et al., 2012, 2014; Mao et al., 2015). These studies reported distinct differences between mucosa and digesta associated bacteria. In the present study, after oral supplementation of Clostridia for 70 days, cows were sacrified to analyze microbial populations in digesta and mucosa by gut compartment as well as in buccal tissue.

**Figure 2:**
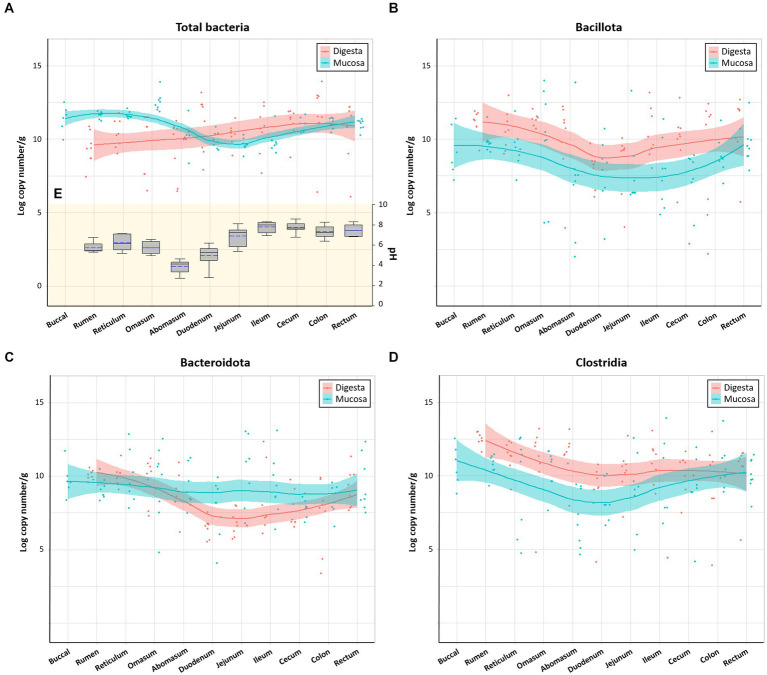
Microbial variations in the gastrointestinal tract of cows. **(A)** Total bacteria, **(B)** Bacillota, **(C)** Bacteroidota, and **(D)** Clostridia. Trend lines with red and blue indicate the variation of quantitative microorganisms in digesta and mucosa, respectively. **(E)** pH variations of the digesta in the intestinal tract. In box plots, square indicates the interquartile range for each data point, and the black and blue lines denote the median and mean values, respectively. The error bars represent the 10th and 90th percentiles and the black circles are outliers.

Bacterial populations in various regions were measured using real-time PCR analysis by analyzing the total copy number of 16S rRNA genes per gram of samples. Data from both control and Clostridial challenge groups were combined to describe the general trend of the microbial variations in luminal and mucosal samples along the gastrointestinal tract (Figure 2). Red and blue lines indicate the trend lines of microbial variation in digesta and mucosa, respectively. Total bacterial density of mucosa of the cows was affected by regional GI sites (Figure 2A). The mucosa-associated bacterial numbers in the rumen and reticulum (11.24–11.91 log copy number/g) decrease in the jejunum to 8.83–10.30 log copy number/g, which can be partly explained by the pH differences of the digesta in different regions (Figure 2E). The pH decrease is due to acid secretion in the abomasum and pancreatic secretions in the duodenum (Antanaitis et al., 2016). The low pH value in the abomasum (pH 2.74–4.62) is correlated to the decrease of microbial populations in the duodenum and jejunum. When comparing the digesta and mucosa-associated bacterial numbers, the mucosa-associated bacteria were higher than digesta in the rumen, reticulum, and omasum while it was lower in the distal part of GI tract from the jejunum. The phylum Bacillota which includes the Clostridia were generally higher in digesta than in mucosa (Figure 2B), which is consistent with a previous report (Mao et al., 2015). Both digesta and mucosa also showed similar trends in that Bacillota decreased in the duodenum to jejunum and then increased again. Likewise, Bacteroidales in digesta showed the same trend, while there was no significant changes in mucosal numbers (Figure 2C). From the duodenum, Bacteroidales in mucosa was higher than in digesta, which is also consistent with a previous study (Mao et al., 2015). Clostridia in both digesta and mucosa decreased in the duodenum and then increased similarly to the Bacillota values (Figure 2D).

### 3.3. Buccal microbiota and pH of digesta by Clostridia challenge

**Figure 3:**
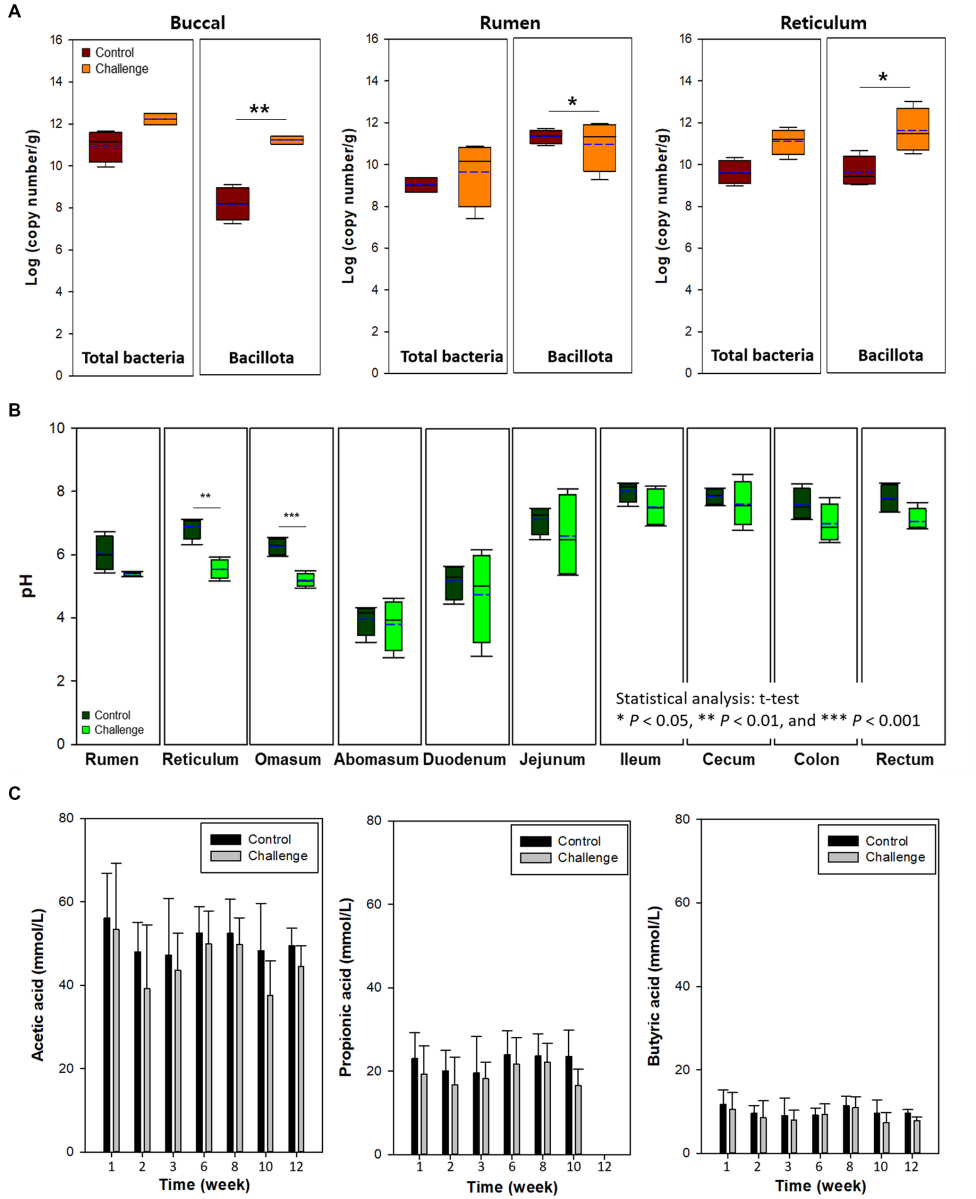
**(A)** Buccal, rumen, and reticulum microbiota. **(B)** pH of digesta in the intestinal tract by clostridial challenge. **(C)** Voletile fatty acids in rumen liquor of cows. In box plots, square indicates the interquartile range for each data point, and the black and blue lines denote the median and mean values, respectively. The error bars represent the 10th and 90^th^ percentiles and the black circles are outliers. Values were significantly different (*p < 0.05, **p < 0.01, ***p < 0.001).

Figure 3A shows the total bacteria and Bacillota in buccal tissues of cow in the control and Clostridial challenge groups. Total bacteria in control and challenge groups was 10.97 and 12.23 log copy number/g (*p* > 0.05). This trend was clearer in the Bacillota that showed bacterial populations were significantly higher in the challenge group than in control (cont: 8.14 log copy number/g; challenge: 11.18 log copy number/g; *p* < 0.01). It seems that the buccal tissue was affected by the Clostridal challenge, as the mouth is directly exposed to Clostridia in the feed. The Clostridial challenge group showed significantly lower pH than control especially in the reticulum (*p* < 0.01) and omasum (*p* < 0.001) (Figure 3B). This might be due to the production of VFAs by the added Clostridia during fermentation (Pearlin et al., 2020). However, the VFA concentrations of the rumen fluid of cows did not show signifant differences between the control and challenge groups (*p* > 0.05, Figure 3C). The highest VFA concentrations were determined as acetate (37.7 – 56.2 mmon/L), followed by propionate (16.8 – 24.0 mmol/L), and butyrate (8.0 – 11.8 mmol/L). Isobutyric acid, valeric acid, isovaleric acid, and acetone were less than 3 mmol/L in both groups. The finding of acetone as a fermentation end product is interesting as it is produced in the acetone-butanol fermentation by solventogenic Clostridia that are also part of the normal commensal Clostridial population in the rumen.

**Figure 4:**
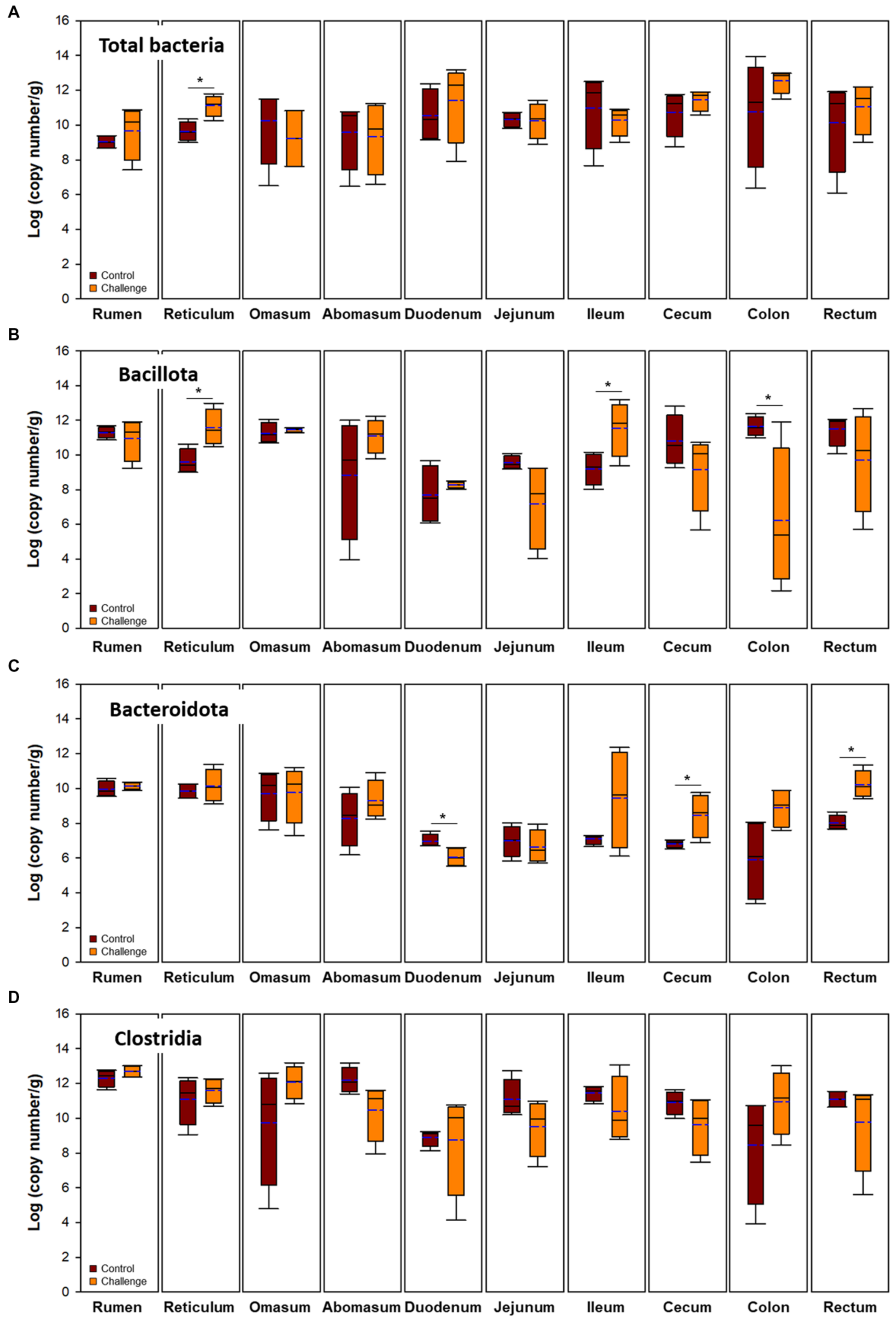
Microbial populations in digesta of intestinal tract. Brown and orange indicate control and clostridial challenge groups, respectively. **(A)** Total bacteria, **(B)** Bacillota, **(C)** Bacteroidota, and **(D)** Clostridia. In box plots, square indicates the interquartile range for each data point, and the black and blue lines denote the median and mean values, respectively. The error bars represent the 10th and 90th percentiles and the black circles are outliers.

Buccal samples can also be used as a proxy for the rumen microbiota of cows as a noninvasive method to estimate rumen microbiota (Young et al., 2020). In the present study, buccal microbiota was similar to the level of total bacteria and Bacillota in the digesta of rumen and reticulum (Figures 4A,B). For example, total bacteria and Bacillota in the rumen and reticulum were higher in the Clostridia challenge group similarly to the buccal microbiota.

### 3.4. Effect of Clostridial challenge on microbial populations

Viable Clostridia in tissue samples of each regions were measured using the plate counting method (Supplementary Figure S2). Similar to the Clostridial challenge data in Supplementary Figure S1, there was an approximately 2 log-difference between control and Clostridial challenge group in rumen samples. However, this gap decreased through out the GI tract and there were large variations between cows.

**Figure 5:**
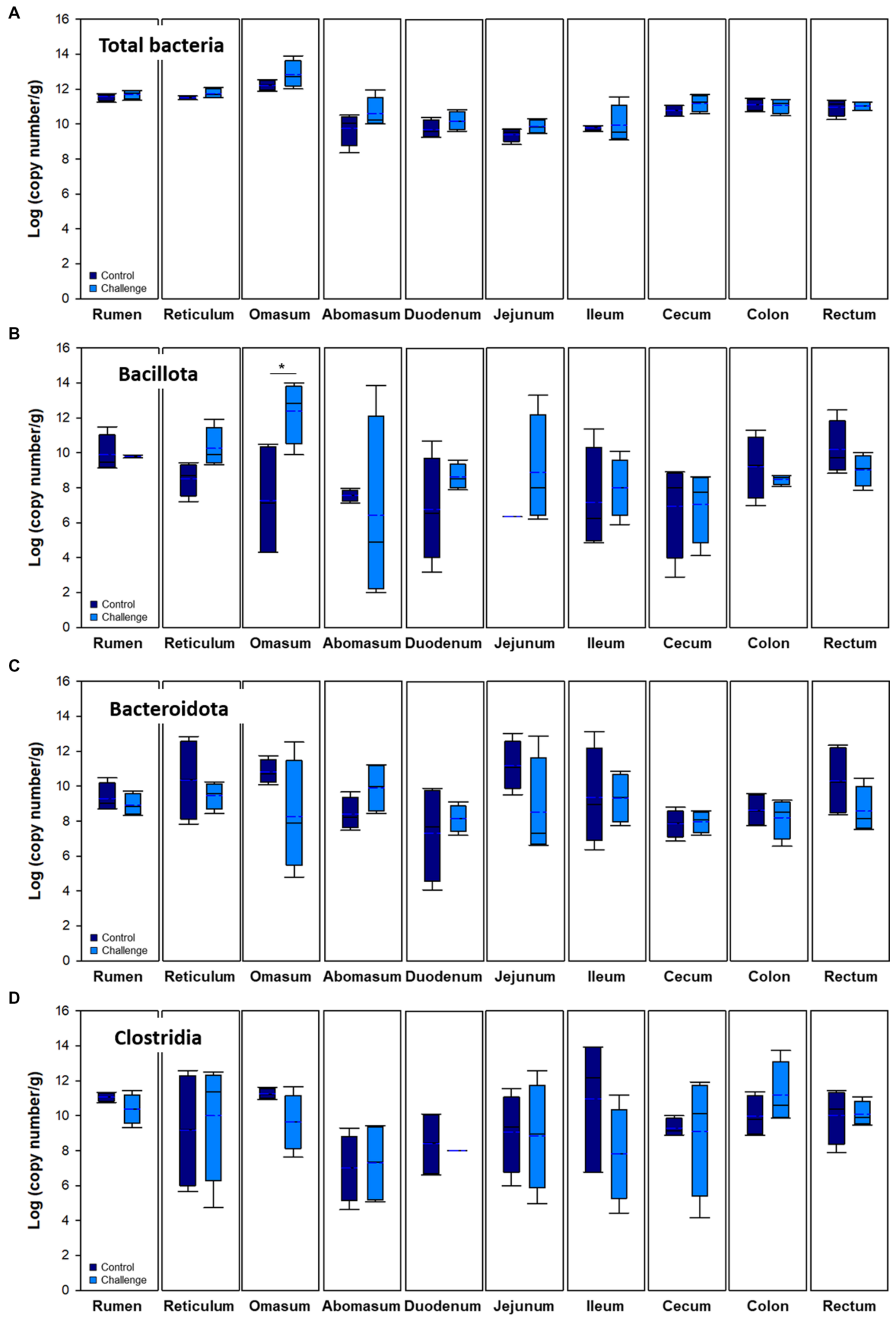
Microbial populations in mucosa of the intestinal tract. Navy and blue indicate control and clostridial challenge groups, respectively. **(A)** Total bacteria, **(B)** Bacillota, **(C)** Bacteroidota, and **(D)** Clostridia. In box plots, square indicates the interquartile range for each data point, and the black and blue lines denote the median and mean values, respectively. The error bars represent the 10 th and 90 th percentiles and the black circles are outliers.

Bacterial populations in digesta and mucosa by Clostridial challenge estimated by qPCR are presented in Figures 4, 5, respectively. Total bacteria were higher in the Clostridial challenge group especially in buccal and foregut tissues, but generally there was no significant difference between the groups (*p* > 0.05) except that bacterial populations in the reticulum digesta samples (control: 9.62 log copy number/g; challenge: 11.12 log copy number/g; *p* < 0.05). In the case of the mucosa, there were no differences in total bacteria. Bacillota tended to be higher in the challenge group but it was reversed in the hindgut sites of the GI tract. In the case of Bacteroidales, the mucosa was not affected by the Clostridial challenge, while significantly higher Bacteroidales in Clostridia challenge group in the cecum and rectum were observed. Unlike the result of Bacillota populations, there were no significant changes in Clostridia and no specific patterns in other clostridial species. In the Clostridial challenge group, the *P. bifermentans* levels were higher in the digesta of the rumen and reticulum, although the difference was not statistically significant (Supplementary Figure S3A, *p* > 0.05). Conversely, we found a statistically significant increase in the levels of *P. bifermentans* in the mucosa of the duodenum and the digesta of the jejunum in the Clostridial challenge group (*p* < 0.05).

### 3.5. 16S rRNA amplicon sequencing

**Figure 6:**
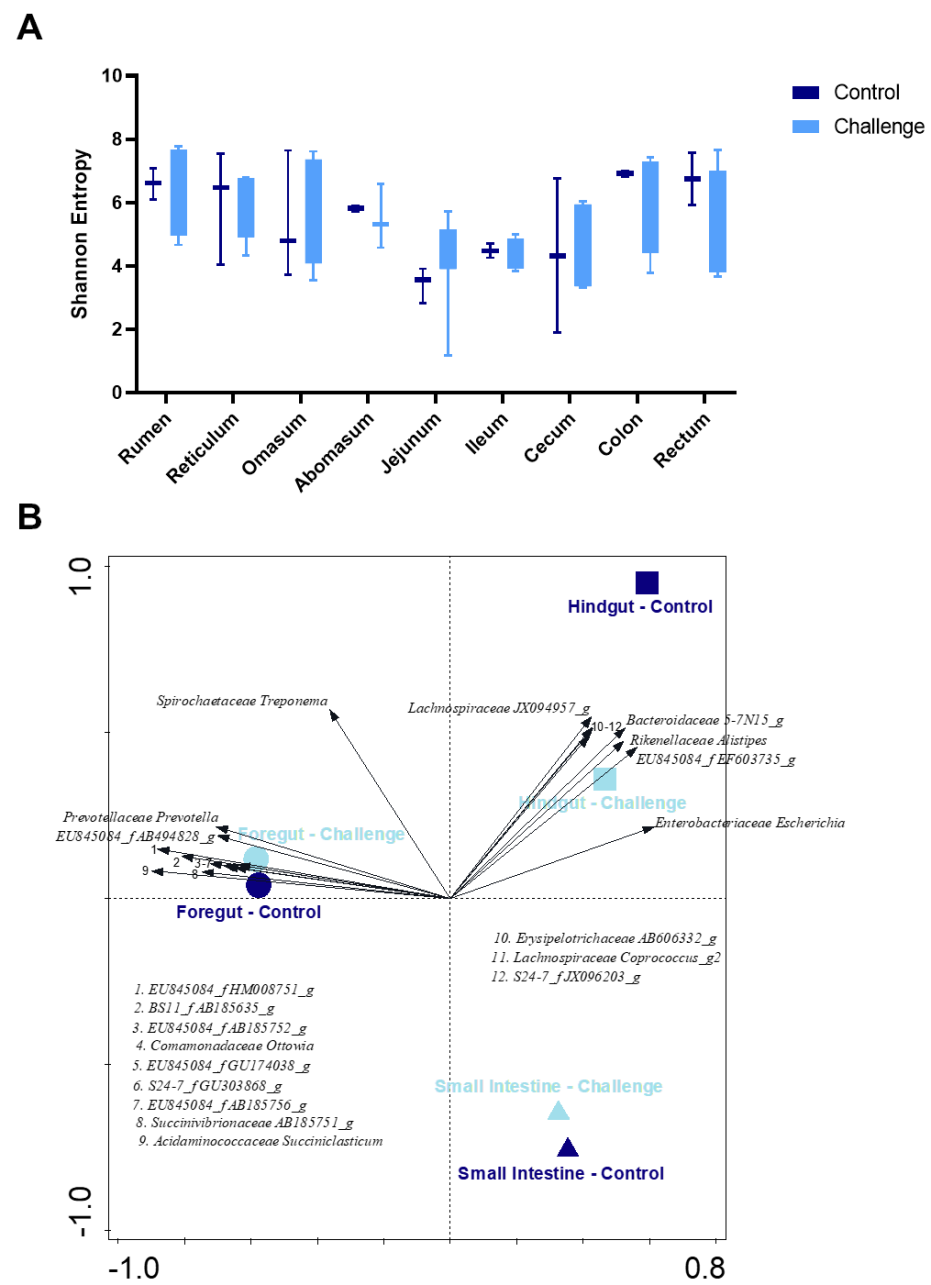
**(A)** Shannon index of α-diversity by gastrointestinal section of control and challenge groups. **(B)** RDA plots of the β-diversity constrained by GI section (foregut, small intestine, and hindgut) × group (control and challenge). 12 taxa included. The 6 genera listed in the fourth quadrant are from the cluster of arrows on the x-axis and are in the same order, top to bottom, as the arrows.

Average Shannon diversity plots of α-diversity are shown in Figure 6A. There was no significant difference between two groups (*p* < 0.05). The influence of GI section (foregut, hindgut, and small intestine) and Clostridial challenge on community-level differentiation (β-diversity) was determined by constrained redundancy analysis (RDA) as shown in Figure 6B showing that 27.2% of the observed variation was accounted for the explanatory variables. The constrained variations resulting from the differences in foregut × challenge group and foregut × control group explain 8.4 and 6.0% of the variation with a significance value of *p* = 0.002.

Previous studies regarding the bacterial microbiota along the GI tract of dairy cattle and preweaned calves reported that the majority belonged to Bacillota (42.2 to 42.7%) followed by Bacteroidales (21.0 to 36.3%), and Pseudomonadota (formerly Proteobacteria) (11.9 to 17.6%) (Malmuthuge et al., 2014; Mao et al., 2015). In general, the rumen bacterial community of one to three-day-old calves was dominated by Pseudomonadota, but as the calves aged it was slowly replaced by Bacteroidales in the rumen and affected the fecal microbiota (Malmuthuge et al., 2014). In this study, control and challenge group microbiota in the overall GI tract were dominated by Bacillota (average 49.46 and 39.41%, respectively), Pseudomonadota (18.75 and 32.14%), and Bacteroidota (21.55 and 20.70%). The other minor (<5%) phyla were dominated by Spirochaetota (formerly Spirochaetes), followed by Mycoplasmatota (formerly Tenericutes), Actinomycetota (formerly Actinobacteria), Fibrobacterota (formerly Fibrobacteres), Lentisphaerota (formerly Lentisphaerae), Candidatus Saccharibacteria (formerly TM7) Verrucomicrobiota (formerly Verrucomicrobia), and others. The relative abundance of Bacteroidota also showed a decreasing trend in the jejunum, ileum, and cecum, which is consistent with the qPCR data (Figures 2, 5).

**Figure 7:**
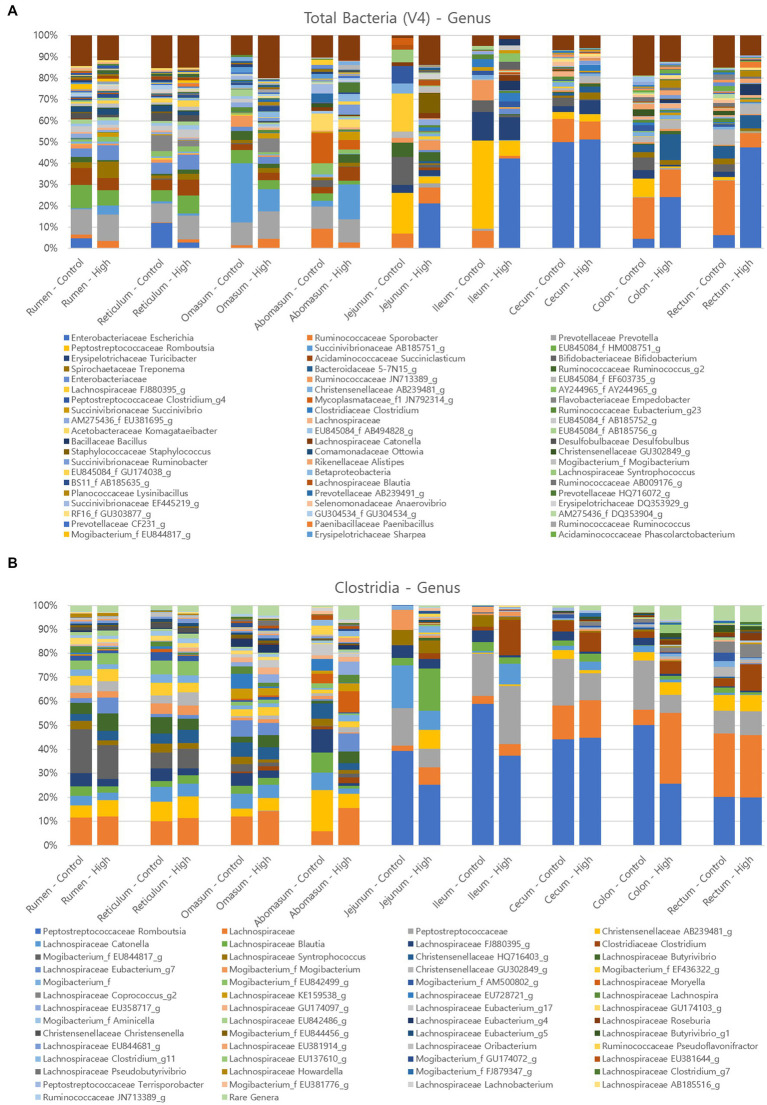
Relative abundance of major microorganisms in control and clostridial challenged cows at the genus level **(A)** Total bacteria, **(B)** Clostridia.

The relative distributions of major ASVs in the control and challenge group cows at the genus level are presented in Figure 7. A total of 372 genera were detected in cow tissues in GI tract. The three predominant genera of the overall GI tract in both control and Clostridial challenge group were *Escherichia* (average 8.60 and 21.01%, respectively), *Prevotella* (4.77 and 5.65%), and *Sporobacter* (9.34 and 5.43%). *Escherichia* was more predominant in the challenge group in the ileum, colon, and rectum (42.31, 24.24, and 47.49%, respectively) compared to the control group (0, 4.44, and 6.30%, respectively). *Lactobacillus* and *Bifidobacterium* are beneficial bacteria regarded as biological indices for the healthy status of animals (Collado and Sanz, 2007). Here, the proportion of *Bifidobacterium* averaged 1.86% in the control group, while in the challenge group they averaged 0.40% and were not detected in rumen, reticulum and proximal jejunum.

As the amplicon sequence variants did not identify any matches with *P. bifermentans*, Clostridia amplicon analysis was conducted to confirm the pattern of Clostridia abundance. The results from the analysis indicated that the relative abundance of *Clostridium* was higher in the challenge group in the jejunum, ileum, cecum, colon, and rectum compared to the control group, as illustrated by the brown color in Figure 7B (control group: 0 to 4.47%; challenge group: 2.41 to 14.63%). These findings suggest that the Clostridial challenge condition may have influenced the colonization of *Clostridium* in the hind gut of the challenged cows, which provide insights into the potential role of Clostridia in the gut microbial communities and host health.

### 3.6. Fecal microbiota

The fecal microbiota of animals has been used to estimate the gut microbiome, although it has been shown that fecal samples do not properly represent the complexity of the gut microbiome (Malmuthuge, 2021). The Clostridial challenge group was initially supplemented by 2 log of total Clostridia more than the control group (Supplementary Figure S1). Accordingly, in fecal samples, there was a significant difference of the viable Clostridial counts between the control and challenge group by 1.63 log (*p* < 0.05, Supplementary Figure S4A).

As a next step, relative abundance of bacterial genera in the fecal samples was determined by the 16S rRNA amplicon sequencing approach. According to the results from a previous study on the fecal bacterial community in several cohorts, though there was a high variability between individual farms, the average relative abundance of fecal microbiota at the phylum level was the highest in Bacillota (59.74%) followed by Bacteroidales (31.54%) (Hagey et al., 2019). Interestingly, Bacteroidales was one of the major bacteria (27˗42%) in this study followed by Bacillota (32˗38%) and Pseudomonadota (20˗23%) (Supplementary Figure S6B). As the relative abundance of Pseudomonadota in cow mucosal tissues (Figure 7A) was relatively high compared to the previous study (Hagey et al., 2019), the abundant Pseudomonadota in fecal samples may reflect the status of gut microbiota. At the genus level (Supplementary Figure S6C), significant differences between control and Clostridial challenge group revealed that the OTU 5-7 N15 belonging to Bacteroidales was the most predominant in the challenge group (24.94%) while it was only 7.98% in the control group.

### 3.7. Immunological responses

**Figure 8:**
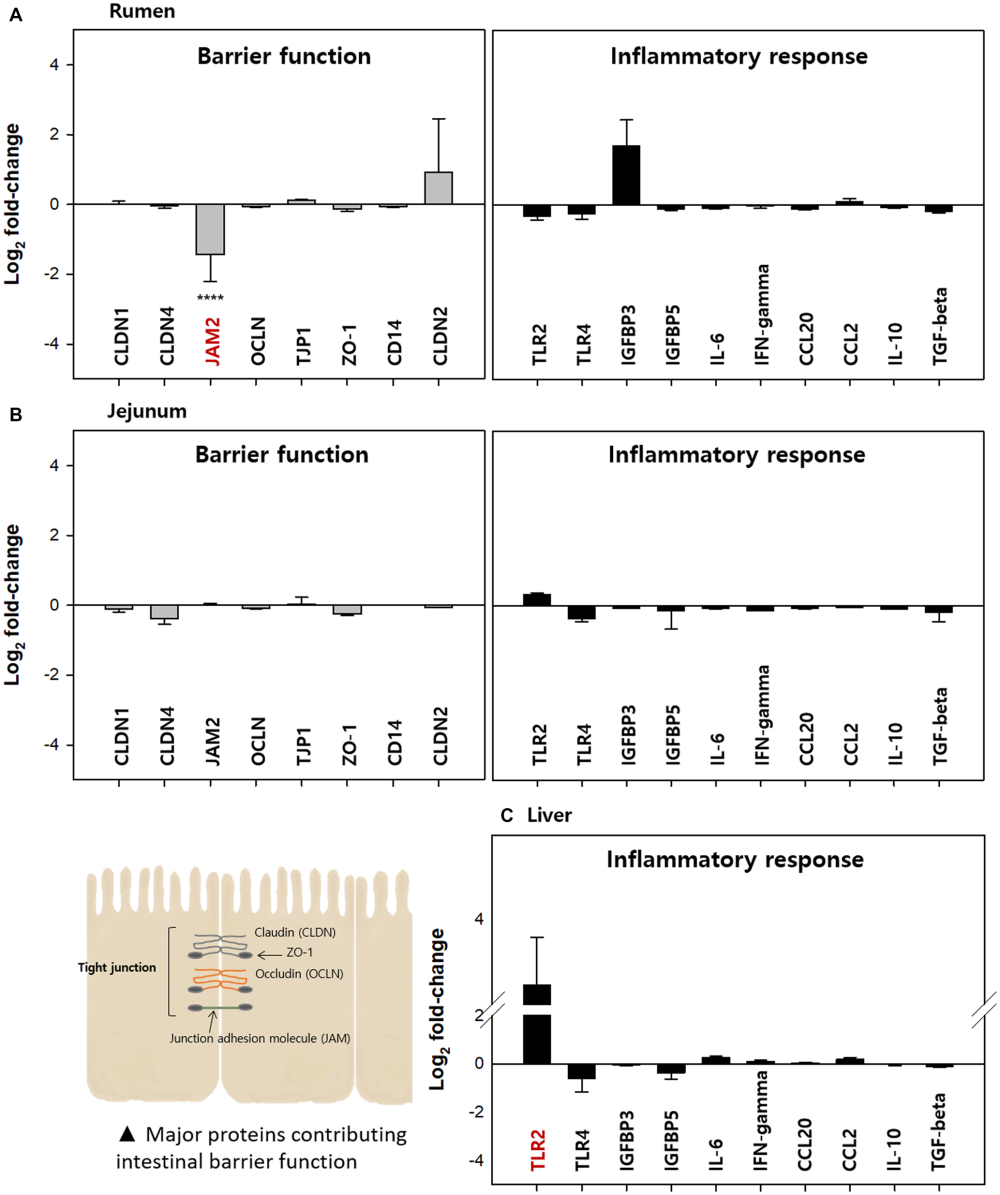
Relative expression of barrier function and immune response-related genes in the **(A)** rumen, **(B)** jejunum, and **(C)** liver of the clostridial challenged cows compared to the control groups A total of eight barrier function related genes encoding CLDN1, CLDN4, JAM2, OCLN, TJP1, ZO-1, CD14, and claudin2 and ten immune-response related genes encoding TLR2, TLR4, IGFBP3, IGFBP5, IL-6, IFN-γ, CCL20, CCL2, IL-10, and TGF-β were analyzed. All tests were performed in triplicate. The value for *JAM1* was significantly different (****p < 0.0001).

In this study, mucosa-associated bacteria in the rumen, jejunum, and liver (a key frontline immune tissue) were used to analyze the relative gene expression related to the barrier function and inflammatory responses (Figure 8). Relative gene expression in the Clostridial challenge group was calculated compared to the control and presented as a log_2_ fold-change (FC). Barrier function genes examined in this study included claudin (CLDN), junction adhesion molecule (JAM), occludin (OCLN), tight junction protein (TJP, ZO-1), and intestinal barrier integrity influencing protein (CD14). Among the genes encoding these proteins, JAM2 encoding gene in rumen tissue was significantly down-regulated by −1.44 log_2_ FC (*p* < 0.0001). This finding implies that a Clostridial challenge could diminish barrier function, like coccidial parasite *Eimeria bovis* decreased the expression of JAM2, OCLN, and TJP1 (Walker et al., 2015). Verification at the protein level is required for confirmation of this finding. Down-regulation of the junction adhesion molecule could lead to a leaky gut, which can induce an inflammatory response (Vetrano et al., 2008). Indeed, expression of one of the inflammatory response related genes, IGFBP3, increased in rumen tissue although it was not significant (*p* > 0.05).

In the jejunum, expression of all genes measured was less than two-fold and there was no significant difference between control and Clostridial challenge groups (*p* > 0.05). This result is likely correlated to the finding that there were no significant changes in microbial populations by Clostridial challenge, which can affect immunological responses. In the case of the liver, barrier function related genes were not examined since there is no intestinal barrier in this tissue. Thus, only inflammatory response related gene expression was analyzed for liver tissue. Here, the toll-like receptor 2 (TLR2) encoding gene was highly up-regulated although it was not significant (3.22 log_2_ FC, *p* > 0.05). The TLR2 plays an important role in the innate immune response to a variety of pathogens (Hayward and Lee, 2014) and acts as an early bactericidal effector molecule of the innate immune system by recognizing pathogens. It is possible that the non-toxigenic commensal Clostridia used in this study could activate the expression of TLR2 in the liver of cows, which means they may be recognized by the immune system like pathogenic bacteria. However, no other inflammatory responses were observed at the transcription level.

While the Clostridial challenge did not clearly affect the mucosa-associated microbial counts especially in the small (duodenum, jejunum, and ileum) and large intestine (cecum, colon, and rectum), it affected the microbial counts in the foregut and microbial diversity throughout the GI tract. It is possible these differences in bacterial diversity could affect immunological responses of the host in a different way. A previous study investigating the mucosal bacteria in the colons of mice associated it with the inflammatory response related gene expressions and suggested mucosa-associated bacteria play a role in stimulating host immune responses (Mulder et al., 2009). Another study also reported that regional differences in the intestinal bacteria of calves could contribute to TLR expression patterns (Malmuthuge et al., 2014).

## 4. Conclusion

To conclude, our study on regional (buccal to rectum) and sample type (mucosa vs. digesta) microbiota revealed that the microbial populations and predominant bacterial profiles in the GI tract of dairy cows can be affected by the challenge with Clostridia even though they are commensal bacteria. Overall, compartmental analysis showed that mucosa-associated bacteria in the foregut tended to decrease in the small intestine affected by pH differences of the digesta. The increase in bacterial populations by Clostridial challenge was obvious in the buccal tissues as well as the anterior part, which is highly likely to the effect of high Clostridial loads in the feed (2 log more Clostridia than the control group). This trend was generally not observed through the distal part of the GI tract and no significant differences in microbial populations were caused by challenge while there were some exceptions in the digesta-associated microbiota. For example, total bacterial populations in the reticulum digesta were higher in the challenge group than in the control group. Firmicutes tended to be high in the challenge group but this trend was reversed in hindgut sites.

Investigating the gut microbiome using 16S rRNA gene sequencing-based approaches also provided valuable insights on the effects of Clostridial challenge. The oral supplementation of commensal Clostridia affected the relative abundance of gut microbiota as well as fecal microbiota. In brief, less *Bifidobacterium* was observed in the mucosa-adhered microbiota and *Escherichia* proportions increased in the challenged group. These observations indicate the possible adverse effect of the *P. bifermentans* to the health status of cows. In particular, transcriptional analysis revealed that there was a down-regulation of barrier function related genes in the rumen by the Clostridial challenge, which may cause leaky gut thereby possibly causing an increased inflammatory responses. While the immune-system did not measurabley respond to the addition of commensal Clostridia and the challenge did not affect the barrier function and immune responses of cows in general, the transition of microbiota and changes in gene expressions suggest the potential immunological risk caused by the commensal Clostridia in feed. Therefore, monitoring, identification, and control of the Clostridia in the dietary component as well as farm environment will be critical for determining cow health and performance.

## Data availability statement

The data presented in the study are deposited in the NCBI repository, accession number PRJNA978622.

## Ethics statement

The animal study was reviewed and approved by The experimental design and animal use procedures were approved by the University of Illinois (Urbana-Champaign) Institutional Animal Care and Use Committee (no. 19036).

## Author contributions

TR, AS, and RM contributed to the conception and design of the study. HK, NK, JT, MJ, and JR performed experiments. JR analyzed and visualized metagenome data. HK wrote the first draft of the manuscript. All authors contributed to the article and approved the submitted version.

## Funding

This study was supported by research funding provided by Arm & Hammer Animal and Food Production. The funder was involved in planning, execution (genome sequencing part), and reviewing of the research.

## Conflict of interest

Authors JT, MJ, JR, TR, and AS were employed by Arm & Hammer Animal and Food Production.

The remaining authors declare that the research was conducted in the absence of any commercial or financial relationships that could be construed as a potential conflict of interest.

## Publisher’s note

All claims expressed in this article are solely those of the authors and do not necessarily represent those of their affiliated organizations, or those of the publisher, the editors and the reviewers. Any product that may be evaluated in this article, or claim that may be made by its manufacturer, is not guaranteed or endorsed by the publisher.
