# Supplementary material for: Effects of dosing non-toxigenic Clostridia on the bacterial populations and immunological responses in the intestinal tract of lactating dairy cows
Source: Front Microbiol. 2023 Jun 15;14:1107964. doi: 10.3389/fmicb.2023.1107964 (PMC10321773; doi:10.3389/fmicb.2023.1107964)
Supplement: Supplementary file 1 [file Data_Sheet_1.docx]

**Supplementary information**

**
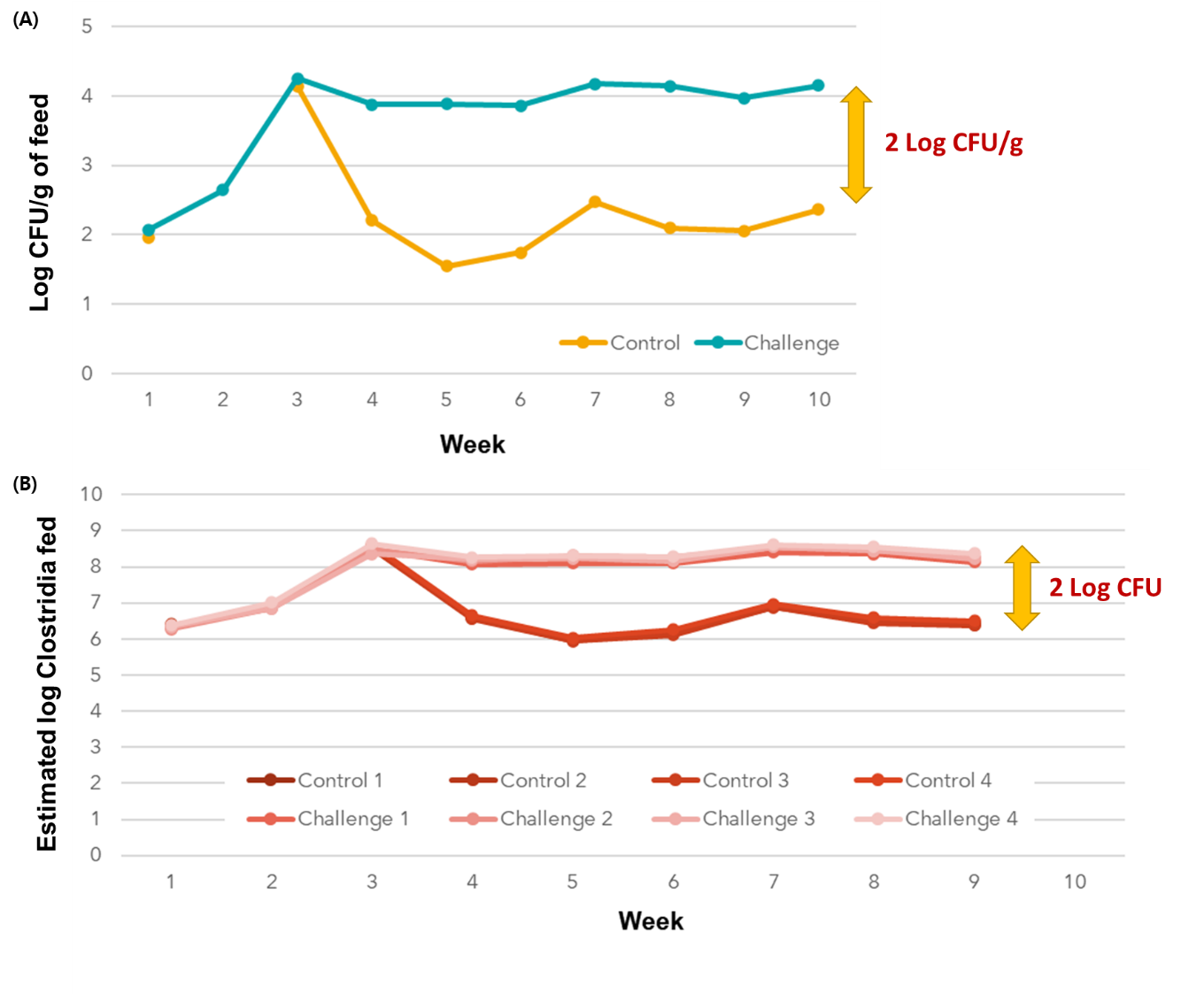
**

**Figure S1.** (A) Weekly Clostridial counts in feed during the experiment of nine weeks. (B) Estimated total Clostridial challenge of dairy cows. The value was calculated as average daily intake (DMI, g) x clostridial counts in feed (CFU/g). Samples at week 2 lost in transit.

**
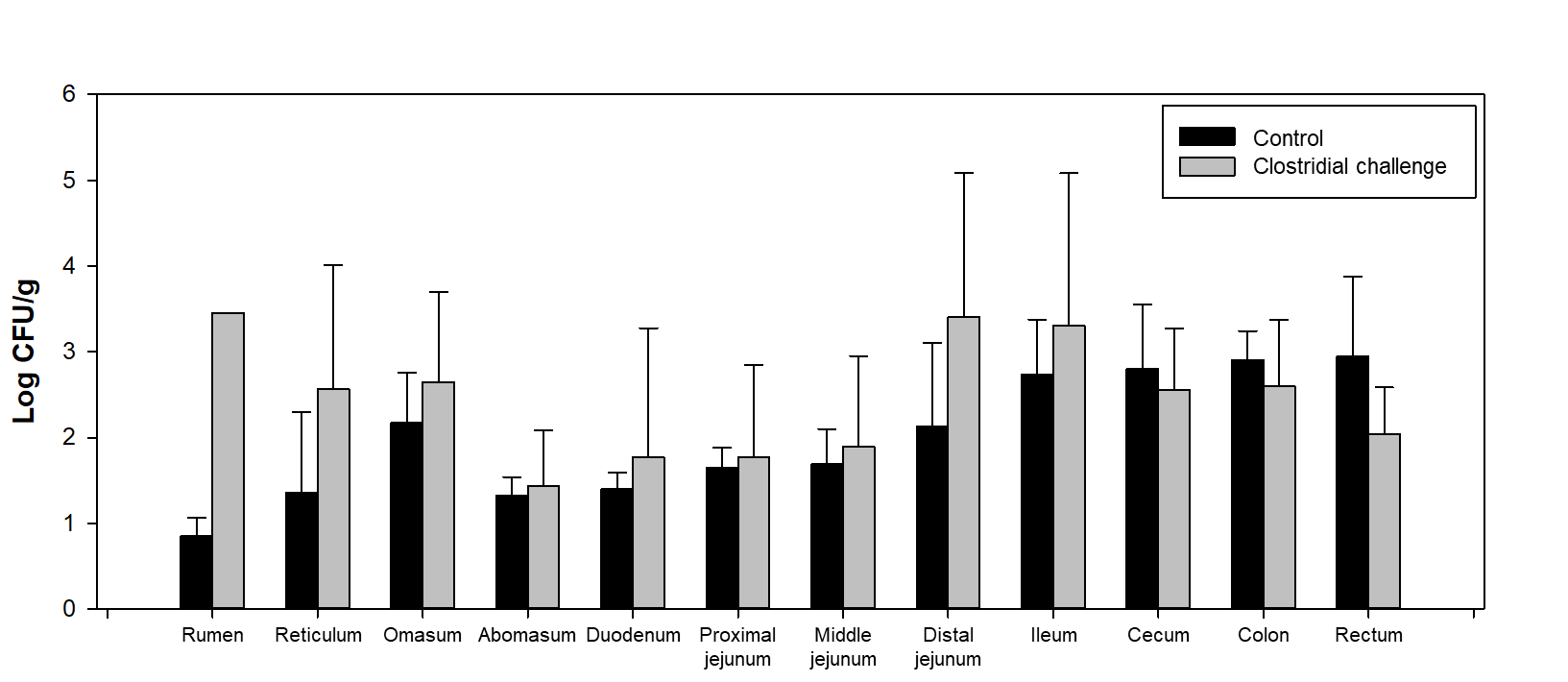
Figure S2.** Viable Clostridial counts in mucosa of GI tract. Black and grey bars indicate control and Clostridial challenged dairy cow.


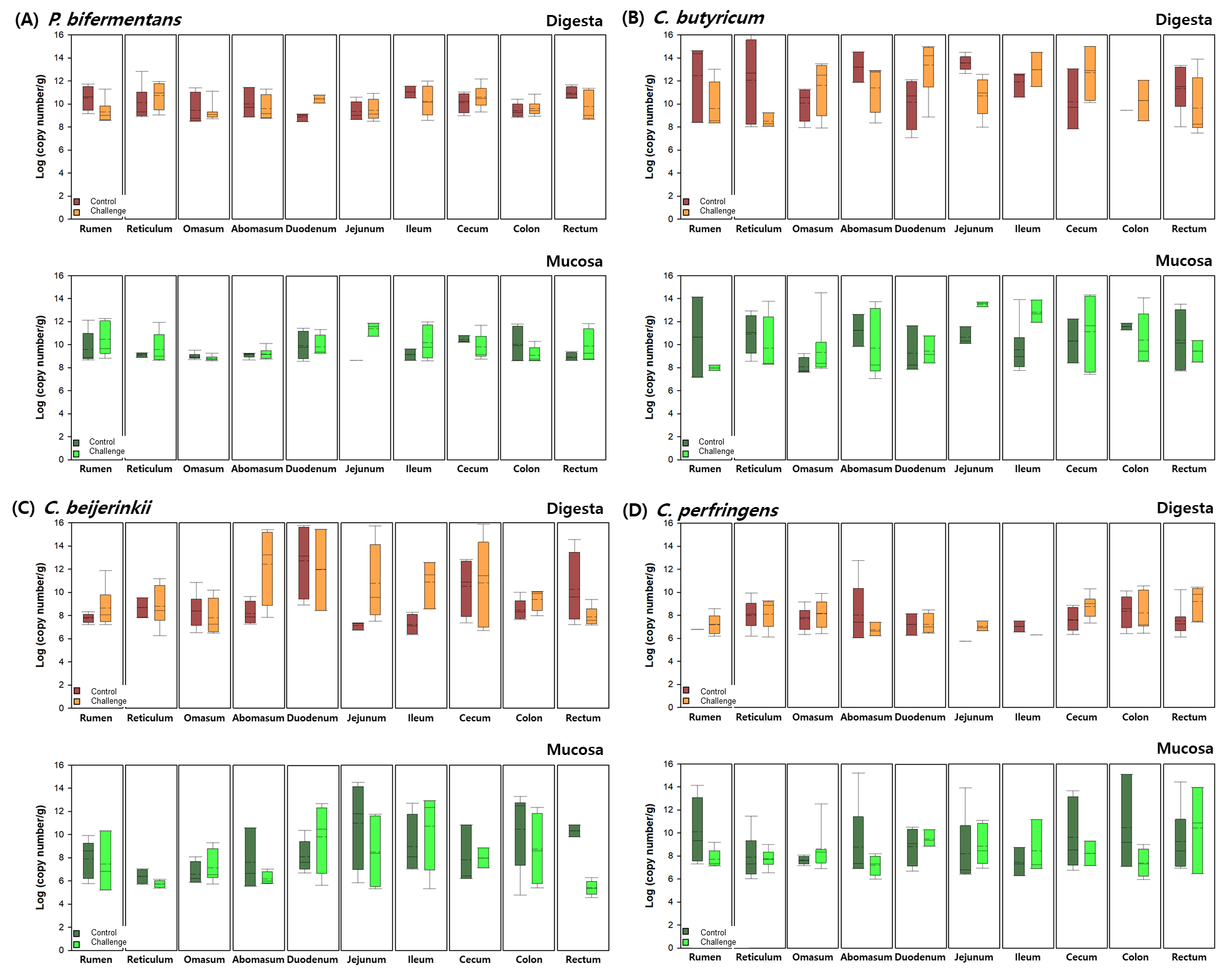


**Figure S3.** Bacterial populations in digesta (brown and orange) and mucosa (green and light green) of the intestinal tract. Brown and green indicate control and orange and light green indicate clostridial challenge groups. (A) *P. bifermentans*, (B) *C. butyricum*, (C) *C. beijerinkii*, and (D) *C. perfringens*. In box plots, square indicates the interquartile range for each data point, and the black and blue lines denote the median and mean values, respectively. The error bars represent the 10^th^ and 90^th^ percentiles and the black circles are outliers.

**
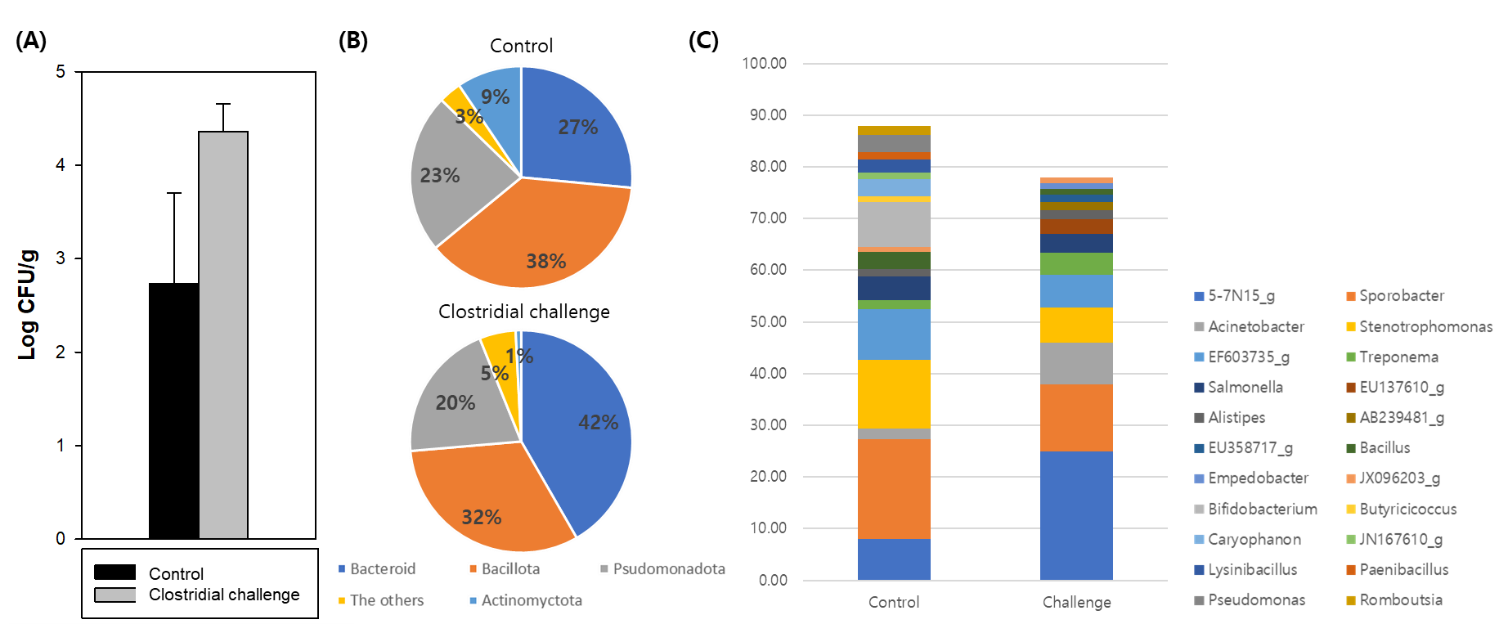
**

**Figure S4.** (A) Viable Clostridial counts in feces of control and Clostridial challenge groups of dairy cows. (B) Relative abundance of microorganisms among control and Clostridial challenged cows at phylum level. (C) Relative abundance of major bacterial taxa (>1%) in control and Clostridial challenged dairy cows at the genus level.

**Table S1.** Primer sequences used to quantitate rumen bacterial taxa

| **Main target** | **Gene** | **Primer sequence (5’ to 3’)** | **Size (bp)** | **Reference** |
| --- | --- | --- | --- | --- |
| Total bacteria | 16S | For: CGGCAACGAGCGCAACCC | 130 | Denman and McSweeney (2006) |
|  |  | Rev: CCATTGTAGCACGTGTGTAGCC |  |  |
| Bacillota | 16S | For: GGAGYATGTGGTTTAATTCGAAGCA | 126 | Guo et al. (2008) |
|  |  | Rev: AGCTGACGACAACCATGCAC |  |  |
| Bacteroidota | 16S | For: GAAGGTCCCCCACATTG | 610 | Reichardt et al. (2014) |
|  |  | Rev: CTTTGAGTTTCACCGTTGCCCG |  |  |
| Clostridia | 16S | For: CGGTGAAATGCGTAGAKATTA | 270 | Hu et al. (2014) |
|  |  | Rev: CGAATTAAACCACATGCTCCG |  |  |
| *C. bifermentans* | 16S | For: CAAGTCGAGCGATCTCT | 564 | Kikuchi et al. (2002) |
|  |  | Rev: CCTGCACTCAAGTTCTCT |  |  |
| *C. butyricum* | 16S | For: TACCGCATGGTACAGCAATT | 1,056 | Kikuchi et al. (2002) |
|  |  | Rev: TCGCGAGGTTGCATCTCAT |  |  |
| *C. beijerinckii* | *nifH* | For: TGACACGATTTTTCATTCTCCA | 448 | Cremonesi et al. (2012) |
|  |  | Rev: TCCATTGCCTTAATGACAGGT |  |  |
| *C. perfringens* | *plc* | For: CCGTTGATAGCGCAGGACA | 219 | Nagpal et al. (2015) |
|  |  | Rev: CCCAACTATGACTCATGCTAGCA |  |  |

**Table S2.** Reference bacterial strains used in this study

| **Main target** | **Bacterial strains used** |  |
| --- | --- | --- |
|  | **Positive control** | **Negative control** |
| Total bacteria | *E. coli* NEB 316 *E. coli* WTT1 *S. epidermidis* ATCC 35984 *C. bifermentans* R59.56.3 *C. beijerinckii* R50.37.1 |  |
| Bacillota | *S. epidermidis* ATCC 35984 *C. bifermentans* R59.56.3 *C. beijerinckii* R50.37.1 | *E. coli* NEB 316 *E. coli* WTT1 |
| Bacteroidota | *P. ruminicola* 23 *B. thetaiotaomicron* 5482 | *S. epidermidis* ATCC 35984 *C. bifermentans* R59.56.3 *C. beijerinckii* R50.37.1 |
| Clostridia | *C. bifermentans* R59.56.3 *C. beijerinckii* R50.37.1 *R. flavefaciens* FD-1 *R. albus* 7 | *E. coli* NEB 316 *E. coli* WTT1 *S. epidermidis* ATCC 35984 |
| *C. bifermentans* | *C. bifermentans* R59.56.3 *C. bifermentans* 1371.G11 *C. bifermentans* 1378.G4 *C. bifermentans* 1378.B3 | *C. beijerinckii* R50.37.1 |
| *C. butyricum* | *C. butyricum* R865.30.4 *C. butyricum* R880.29.2 | *C. beijerinckii* R50.37.1 |
| *C. beijerinckii* | *C. beijerinckii* R50.37.1 *C. beijerinckii* 1371.F11 *C. beijerinckii* 1370.A2 *C. beijerinckii* 1378.F9 | *C. bifermentans* R59.56.3 |
| *C. perfringens* | *C. perfringens* R880.30.1 *C. perfringens* R865.31.5 | *C. beijerinckii* R50.37.1 |

**Table S3.** Real-time qPCR standard curves for DNA quantification of the samples

| **Target organisms** | **Equation** | **R-square** |
| --- | --- | --- |
| Total bacteria | y = -4.9672x + 64.319 | R² = 0.9613 |
| Bacillota | y = -2.3926x + 35.893 | R² = 0.9998 |
| Bacteroidota | y = -3.5296x + 46.954 | R² = 0.9804 |
| Clostridia | y = -3.0100x + 44.782 | R² = 0.9977 |
| *C. bifermentans* | y = -8.2556x + 93.130 | R² = 0.9206 |
| *C. butyricum* | y = -3.9130x + 61.960 | R² = 0.9437 |
| *C. beijerinckii* | y = -3.4363x + 52.707 | R² = 0.9944 |
| *C. perfringens* | y = -3.5593x + 53.515 | R² = 0.9978 |

**Table S4.** Primer sequences targeting barrier function, inflammatory response, and housekeeping genes in the rumen and jejunum mucosa and liver

| **Gene** | **Primer sequence (5’-3’)** | **Size (bp)** | **Reference** |
| --- | --- | --- | --- |
| **Barrier function** | | | |
| CLDN1 | For: GGCATCCTGCTGGGACTAATAG  Rev: CAGCCATCCGCATCTTCTGT | 100 | Minuti et al. 2015 |
| CLDN4 | For: CCCCAGCCAGCAACTACGT  Rev: TCACAGATTGCAGTGAGCTCAGT | 103 | Minuti et al. 2015 |
| JAM2 | For: CCCCATCGGAACAAGGTCAA  Rev: GACATCGCAGCTCTACCACA | 129 | Walker et al. 2014 |
| OCLN | For: GCCATTTTCGCCTGTGTTG  Rev: CCAAAGGCACTTCCTGCATAA | 101 | Minuti et al. 2015 |
| TJP1 | For: GCACATAGGATCCCTGAACCA  Rev: TGCTTCCGGTAGTACTCCTCATC | 107 | Minuti et al. 2015 |
| ZO-1 | For: AGTCATTGCTGCTGGATTTACA  Rev: AATCACCCACATCGGATTCT | 161 | Hu et al. 2018 |
| CD14 | For: CACCACATTGCACACCTGTT  Rev: CACCACATTGCACACCTGTT | 124 | Stefanska et al. 2018 |
| Claudin2 | For: GCAAACAGGCTCCGAAGATAC  Rev: GAGATGATGCCCAAGTACAGAG |  | Zhang et al. 2015 |
| **Inflammatory response** | | | |
| TLR2 | For: CTGGCAAGTGGATTATCGACAA  Rev: TACTTGCACCACTCGCTCTTCA | 102 | Jacometo et al. 2015 |
| TLR4 | For: TGCGTACAGGTTGTTCCTAACATT  Rev: TAGTTAAAGCTCAGGTCCAGCATCT | 109 | Jacometo et al. 2015 |
| IGFBP3 | For: GCGCCCTTACCTGCTACC  Rev: CAGCCTGGTTCTCTGTGCT | 86 | Grala et al. 2014 |
| IGFBP5 | For: GTCCAAGTTCGTGGGAGGAG  Rev: AGGGCCCCTGCTCAGATTTC | 89 | McCann et al. 2016 |
| IL-6 | For: TCCTTGCTGCTTTCACACTC  Rev: CACCCCAGGCAGACTACTTC | 129 | Zhan et al. 2019 |
| IFN-gamma | For: TCCGGCCTAACTCTCTCCTAA  Rev: CCCTGGCCATAAGAACCAGAA | 100 | Zhou et al. 2018 |
| CCL20  (MIP-3-alpha) | For: TTCGACTGCTGTCTCCGATA  Rev: GCACAACTTGTTTCACCCACT | 172 | Zhan et al. 2019 |
| CCL2 (MCP-1) | For: TGCAGACCCCAAGCAGAAA  Rev: AGAGGGCAGTTAGGGAAAGC |  | Sakumoto et al. 2017 |
| IL-10 | For: GAAGGACCAACTGCACAGCTT  Rev: AAAACTGGATCATTTCCGACAAG | 98 | Zhou et al. 2018 |
| TGF-beta | For: CTTTCTTCAAATGCAGCATTGG  Rev: GGGTCTGGGTGATACAACGAA |  | Elweza et al. 2017 |
| **Housekeeping genes** | | | |
| CMTM6 | For: TTCACTTTGACACATGACAATACCA  Rev: CACGGAGCATAAAGGAGAACTCA | 103 | Minuti et al., 2015 |
| ERC1 | For: CCTCCCATTCCGGTCAAAG  Rev: GTCTGATGTACAACTTGAGCTTGCTT | 105 | Naeem et al., 2012 |
| MRPL39 | For: AGGTTCTCTTTTGTTGGCATCC  Rev: TTGGTCAGAGCCCCAGAAGT | 101 | Bionaz and Loor, 2007 |

**Table S5.** Taxonomic string of all genera in the V4 analysis.

| Kingdom | Phylum | Class | Order | Family | Genus | Label |
| --- | --- | --- | --- | --- | --- | --- |
| Bacteria | Proteobacteria | Gammaproteobacteria | Enterobacteriales | Enterobacteriaceae | Escherichia | Enterobacteriaceae Escherichia |
| Bacteria | Bacteroidetes | Bacteroidia | Bacteroidales | Prevotellaceae | Prevotella | Prevotellaceae Prevotella |
| Bacteria | Firmicutes | Clostridia | Clostridiales | Ruminococcaceae | Sporobacter | Ruminococcaceae Sporobacter |
| Bacteria | Bacteroidetes | Bacteroidia | Bacteroidales | Bacteroidaceae | 5-7N15_g | Bacteroidaceae 5-7N15_g |
| Bacteria | Bacteroidetes | Bacteroidia | Bacteroidales | EU845084_f | HM008751_g | EU845084_f HM008751_g |
| Bacteria | Proteobacteria | Gammaproteobacteria | Aeromonadales | Succinivibrionaceae | AB185751_g | Succinivibrionaceae AB185751_g |
| Bacteria | Firmicutes | Clostridia | Clostridiales | Peptostreptococcaceae | Romboutsia | Peptostreptococcaceae Romboutsia |
| Bacteria | Firmicutes | Negativicutes | Acidaminococcales | Acidaminococcaceae | Succiniclasticum | Acidaminococcaceae Succiniclasticum |
| Bacteria | Bacteroidetes | Bacteroidia | Bacteroidales | EU845084_f | EF603735_g | EU845084_f EF603735_g |
| Bacteria | Spirochaetes | Spirochaetes_c | Spirochaetales | Spirochaetaceae | Treponema | Spirochaetaceae Treponema |
| Bacteria | Firmicutes | Clostridia | Clostridiales | Ruminococcaceae | Ruminococcus_g2 | Ruminococcaceae Ruminococcus_g2 |
| Bacteria | Bacteroidetes | Bacteroidia | Bacteroidales | AY244965_f | AY244965_g | AY244965_f AY244965_g |
| Bacteria | Firmicutes | Clostridia | Clostridiales | Ruminococcaceae | JN713389_g | Ruminococcaceae JN713389_g |
| Bacteria | Actinobacteria | Actinobacteria_c | Bifidobacteriales | Bifidobacteriaceae | Bifidobacterium | Bifidobacteriaceae Bifidobacterium |
| Bacteria | Firmicutes | Erysipelotrichi | Erysipelotrichales | Erysipelotrichaceae | Turicibacter | Erysipelotrichaceae Turicibacter |
| Bacteria | Firmicutes | Clostridia | Clostridiales | Christensenellaceae | AB239481_g | Christensenellaceae AB239481_g |
| Bacteria | Firmicutes | Clostridia | Clostridiales | Lachnospiraceae |  | Lachnospiraceae |
| Bacteria | Proteobacteria | Gammaproteobacteria | Aeromonadales | Succinivibrionaceae | Succinivibrio | Succinivibrionaceae Succinivibrio |
| Bacteria | Bacteroidetes | Bacteroidia | Bacteroidales | EU845084_f | AB185752_g | EU845084_f AB185752_g |
| Bacteria | Tenericutes | Mollicutes | AM275436_o | AM275436_f | EU381695_g | AM275436_f EU381695_g |
| Bacteria | Bacteroidetes | Bacteroidia | Bacteroidales | EU845084_f | AB185756_g | EU845084_f AB185756_g |
| Bacteria | Bacteroidetes | Bacteroidia | Bacteroidales | EU845084_f | AB494828_g | EU845084_f AB494828_g |
| Bacteria | Firmicutes | Clostridia | Clostridiales | Ruminococcaceae | Eubacterium_g23 | Ruminococcaceae Eubacterium_g23 |
| Bacteria | Bacteroidetes | Bacteroidia | Bacteroidales | Rikenellaceae | Alistipes | Rikenellaceae Alistipes |
| Bacteria | Bacteroidetes | Bacteroidia | Bacteroidales | EU845084_f | GU174038_g | EU845084_f GU174038_g |
| Bacteria | Firmicutes | Clostridia | Clostridiales | Ruminococcaceae | AB009176_g | Ruminococcaceae AB009176_g |
| Bacteria | Tenericutes | Mollicutes | Mycoplasmatales | Mycoplasmataceae_f1 | JN792314_g | Mycoplasmataceae_f1 JN792314_g |
| Bacteria | Firmicutes | Negativicutes | Selenomonadales | Selenomonadaceae | Anaerovibrio | Selenomonadaceae Anaerovibrio |
| Bacteria | Proteobacteria | Deltaproteobacteria | Desulfobulbaceae_o | Desulfobulbaceae | Desulfobulbus | Desulfobulbaceae Desulfobulbus |
| Bacteria | Bacteroidetes | Bacteroidia | Bacteroidales | GU304534_f | GU304534_g | GU304534_f GU304534_g |
| Bacteria | Firmicutes | Clostridia | Clostridiales | Clostridiaceae | Clostridium | Clostridiaceae Clostridium |
| Bacteria | Proteobacteria | Gammaproteobacteria | Aeromonadales | Succinivibrionaceae | Ruminobacter | Succinivibrionaceae Ruminobacter |
| Bacteria | Bacteroidetes | Bacteroidia | Bacteroidales | RF16_f | GU303877_g | RF16_f GU303877_g |
| Bacteria | Firmicutes | Clostridia | Clostridiales | Christensenellaceae | GU302849_g | Christensenellaceae GU302849_g |
| Bacteria | Proteobacteria | Gammaproteobacteria | Aeromonadales | Succinivibrionaceae | EF445219_g | Succinivibrionaceae EF445219_g |
| Bacteria | Firmicutes | Clostridia | Clostridiales | Mogibacterium_f | Mogibacterium | Mogibacterium_f Mogibacterium |
| Bacteria | Firmicutes | Clostridia | Clostridiales | Lachnospiraceae | Blautia | Lachnospiraceae Blautia |
| Bacteria | Proteobacteria | Betaproteobacteria | Burkholderiales | Comamonadaceae | Ottowia | Comamonadaceae Ottowia |
| Bacteria | Bacteroidetes | Bacteroidia | Bacteroidales | BS11_f | AB185635_g | BS11_f AB185635_g |
| Bacteria | Proteobacteria | Gammaproteobacteria | Enterobacteriales | Enterobacteriaceae |  | Enterobacteriaceae |
| Bacteria | Firmicutes | Clostridia | Clostridiales | Peptostreptococcaceae | Clostridium_g4 | Peptostreptococcaceae Clostridium_g4 |
| Bacteria | Proteobacteria | Betaproteobacteria | __ |  |  | Betaproteobacteria |
| Bacteria | Bacteroidetes | Bacteroidia | Bacteroidales | Prevotellaceae | CF231_g | Prevotellaceae CF231_g |
| Bacteria | Firmicutes | Negativicutes | Acidaminococcales | Acidaminococcaceae | Phascolarctobacterium | Acidaminococcaceae Phascolarctobacterium |
| Bacteria | Bacteroidetes | Bacteroidia | Bacteroidales | Prevotellaceae | AB239491_g | Prevotellaceae AB239491_g |
| Bacteria | Firmicutes | Erysipelotrichi | Erysipelotrichales | Erysipelotrichaceae | DQ353929_g | Erysipelotrichaceae DQ353929_g |
| Bacteria | Firmicutes | Clostridia | Clostridiales | Lachnospiraceae | Catonella | Lachnospiraceae Catonella |
| Bacteria | Firmicutes | Clostridia | Clostridiales | Lachnospiraceae | FJ880395_g | Lachnospiraceae FJ880395_g |
| Bacteria | Firmicutes | Negativicutes | Selenomonadales | Selenomonadaceae | Mitsuokella | Selenomonadaceae Mitsuokella |
| Bacteria | Proteobacteria | Epsilonproteobacteria | Campylobacterales | Campylobacteraceae | Campylobacter | Campylobacteraceae Campylobacter |
| Bacteria | Firmicutes | Clostridia | Clostridiales | Mogibacterium_f | EU844817_g | Mogibacterium_f EU844817_g |
| Bacteria | Fibrobacteres | Fibrobacteria | Fibrobacterales | Fibrobacteraceae | Fibrobacter | Fibrobacteraceae Fibrobacter |
| Bacteria | Firmicutes | Clostridia | Clostridiales | Mogibacterium_f | EF436322_g | Mogibacterium_f EF436322_g |
| Bacteria | Bacteroidetes | Bacteroidia | Bacteroidales | Prevotellaceae | HQ716072_g | Prevotellaceae HQ716072_g |
| Bacteria | Tenericutes | Mollicutes | AM275436_o | AM275436_f | DQ353904_g | AM275436_f DQ353904_g |
| Bacteria | Firmicutes | Clostridia | Clostridiales | Lachnospiraceae | Coprococcus_g2 | Lachnospiraceae Coprococcus_g2 |
| Bacteria | Lentisphaerae | CP010904_c | EF436358_o | EF436358_f | EF436358_g | EF436358_f EF436358_g |
| Bacteria | Proteobacteria | Alphaproteobacteria | Rhodospirillales | Acetobacteraceae | Komagataeibacter | Acetobacteraceae Komagataeibacter |
| Bacteria | Firmicutes | Clostridia | Clostridiales | Ruminococcaceae | Ruminococcus | Ruminococcaceae Ruminococcus |
| Bacteria | Verrucomicrobia | Verrucomicrobiae | Verrucomicrobiales | Akkermansiaceae | HM630201_g | Akkermansiaceae HM630201_g |
| Bacteria | Firmicutes | Clostridia | Clostridiales | Lachnospiraceae | Eubacterium_g4 | Lachnospiraceae Eubacterium_g4 |
| Bacteria | Firmicutes | Erysipelotrichi | Erysipelotrichales | Erysipelotrichaceae | AB606332_g | Erysipelotrichaceae AB606332_g |
| Bacteria | Firmicutes | Clostridia | Clostridiales | Lachnospiraceae | Syntrophococcus | Lachnospiraceae Syntrophococcus |
| Bacteria | Firmicutes | Clostridia | Clostridiales | Lachnospiraceae | Butyrivibrio | Lachnospiraceae Butyrivibrio |
| Bacteria | Firmicutes | Clostridia | Clostridiales | Lachnospiraceae | EU842486_g | Lachnospiraceae EU842486_g |
| Bacteria | Firmicutes | Clostridia | Clostridiales | Ruminococcaceae | Saccharofermentans | Ruminococcaceae Saccharofermentans |
| Bacteria | SR1 | CP006913_c | CP006913_o | CP006913_f | GU410548_g | CP006913_f GU410548_g |
| Bacteria | Bacteroidetes | Bacteroidia | Bacteroidales | Prevotellaceae | GU304053_g | Prevotellaceae GU304053_g |
| Bacteria | Firmicutes | Erysipelotrichi | Erysipelotrichales | Erysipelotrichaceae |  | Erysipelotrichaceae |
| Bacteria | Bacteroidetes | Bacteroidia | Bacteroidales | EU845084_f | EU845084_g | EU845084_f EU845084_g |
| Bacteria | Bacteroidetes | Bacteroidia | Bacteroidales | EU845084_f | EU461334_g | EU845084_f EU461334_g |
| Bacteria | Bacteroidetes | Flavobacteria | Flavobacteriales | Flavobacteriaceae | Empedobacter | Flavobacteriaceae Empedobacter |
| Bacteria | Bacteroidetes | Bacteroidia | Bacteroidales | Bacteroidaceae | EU464174_g | Bacteroidaceae EU464174_g |
| Bacteria | Firmicutes | Clostridia | Clostridiales | Ruminococcaceae | Pseudoflavonifractor | Ruminococcaceae Pseudoflavonifractor |
| Bacteria | Firmicutes | Clostridia | Clostridiales | Lachnospiraceae | Eubacterium_g7 | Lachnospiraceae Eubacterium_g7 |
| Bacteria | Firmicutes | Clostridia | Clostridiales | Ruminococcaceae |  | Ruminococcaceae |
| Bacteria | Firmicutes | Bacilli | Lactobacillales | Lactobacillaceae | Lactobacillus | Lactobacillaceae Lactobacillus |
| Bacteria | Bacteroidetes | Bacteroidia | Bacteroidales | S24-7_f | GU303868_g | S24-7_f GU303868_g |
| Bacteria | Bacteroidetes | Bacteroidia | Bacteroidales | Bacteroidaceae | Bacteroides | Bacteroidaceae Bacteroides |
| Bacteria | Bacteroidetes | Bacteroidia | Bacteroidales |  |  | Bacteroidales |
| Bacteria | Bacteroidetes | Bacteroidia | Bacteroidales | EU845084_f | GU302773_g | EU845084_f GU302773_g |
| Bacteria | Bacteroidetes | Bacteroidia | Bacteroidales | RF16_f | EU843998_g | RF16_f EU843998_g |
| Bacteria | Firmicutes | Clostridia | Clostridiales | Peptostreptococcaceae | Paeniclostridium | Peptostreptococcaceae Paeniclostridium |
| Bacteria | Firmicutes | Clostridia | Clostridiales | Ruminococcaceae | AB185816_g | Ruminococcaceae AB185816_g |
| Bacteria | Tenericutes | Mollicutes | Acholeplasmatales | Acholeplasmataceae | Acholeplasma_g2 | Acholeplasmataceae Acholeplasma_g2 |
| Bacteria | Proteobacteria | Deltaproteobacteria | Desulfovibrionales | Desulfovibrionaceae | Desulfovibrio | Desulfovibrionaceae Desulfovibrio |
| Bacteria | Firmicutes | Clostridia | Clostridiales | Lachnospiraceae | Oribacterium | Lachnospiraceae Oribacterium |
| Bacteria | Saccharibacteria_TM7 | Saccharimonas_c | Saccharimonas_o | Saccharimonas_f | AJ400239_g | Saccharimonas_f AJ400239_g |
| Bacteria | Proteobacteria | Betaproteobacteria | Neisseriales | Neisseriaceae | Snodgrassella | Neisseriaceae Snodgrassella |
| Bacteria | Bacteroidetes | Bacteroidia | Bacteroidales | S24-7_f | JN167610_g | S24-7_f JN167610_g |
| Bacteria | Firmicutes | Clostridia | Clostridiales | Christensenellaceae | HQ716403_g | Christensenellaceae HQ716403_g |
| Bacteria | Bacteroidetes | Bacteroidia | Bacteroidales | Prevotellaceae | Alloprevotella | Prevotellaceae Alloprevotella |
| Bacteria | Firmicutes | Clostridia | Clostridiales | Christensenellaceae | Christensenella | Christensenellaceae Christensenella |
| Bacteria | Firmicutes | Bacilli | Bacillales | Staphylococcaceae | Staphylococcus | Staphylococcaceae Staphylococcus |
| Bacteria | Firmicutes | Clostridia | Clostridiales | Lachnospiraceae | GU174103_g | Lachnospiraceae GU174103_g |
| Bacteria | Firmicutes | Clostridia | Clostridiales | Ruminococcaceae | EU794292_g | Ruminococcaceae EU794292_g |
| Bacteria | Firmicutes | Clostridia | Clostridiales | Ruminococcaceae | Oscillibacter | Ruminococcaceae Oscillibacter |
| Bacteria | Tenericutes | Mollicutes | AM275436_o | AM275436_f | AM275436_g | AM275436_f AM275436_g |
| Bacteria | Proteobacteria | Gammaproteobacteria | Cardiobacteriales | Cardiobacteriaceae | Suttonella | Cardiobacteriaceae Suttonella |
| Bacteria | Firmicutes | Bacilli | Bacillales | Planococcaceae | Lysinibacillus | Planococcaceae Lysinibacillus |
| Bacteria | Firmicutes | Clostridia | Clostridiales | Lachnospiraceae | Eubacterium_g5 | Lachnospiraceae Eubacterium_g5 |
| Bacteria | Firmicutes | Bacilli | Lactobacillales | Streptococcaceae | Streptococcus | Streptococcaceae Streptococcus |
| Bacteria | Firmicutes | Clostridia | Clostridiales | Mogibacterium_f |  | Mogibacterium_f |
| Bacteria | Tenericutes | Mollicutes | AM275436_o |  |  | AM275436_o |
| Bacteria | Firmicutes | Bacilli | Bacillales | Bacillaceae | Bacillus | Bacillaceae Bacillus |
| Bacteria | Proteobacteria | Deltaproteobacteria | Desulfovibrionales | Desulfovibrionaceae | DQ815907_g | Desulfovibrionaceae DQ815907_g |
| Bacteria | Firmicutes | Clostridia | Clostridiales | Lachnospiraceae | Lachnospira | Lachnospiraceae Lachnospira |
| Bacteria | Proteobacteria | Betaproteobacteria | Neisseriales | Neisseriaceae | Simonsiella | Neisseriaceae Simonsiella |
| Bacteria | Firmicutes | Erysipelotrichi | Erysipelotrichales | Erysipelotrichaceae | Sharpea | Erysipelotrichaceae Sharpea |
| Bacteria | Firmicutes | Erysipelotrichi | Erysipelotrichales | Erysipelotrichaceae | Dielma | Erysipelotrichaceae Dielma |
| Bacteria | Proteobacteria | Alphaproteobacteria | Rhodospirillales | Rhodospirillaceae | LARJ_g | Rhodospirillaceae LARJ_g |
| Bacteria | Firmicutes | Bacilli | Lactobacillales | Enterococcaceae | Enterococcus | Enterococcaceae Enterococcus |
| Bacteria | Firmicutes | Clostridia | Clostridiales | Mogibacterium_f | EU844456_g | Mogibacterium_f EU844456_g |
| Bacteria | Bacteroidetes | Bacteroidia | Bacteroidales | Porphyromonadaceae | EU465694_g | Porphyromonadaceae EU465694_g |
| Bacteria | Bacteroidetes | Bacteroidia | Bacteroidales | S24-7_f | EF406773_g | S24-7_f EF406773_g |
| Bacteria | Cyanobacteria | Vampirovibrio_c | FR888536_o | FR888536_f | FR888536_g | FR888536_f FR888536_g |
| Bacteria | Tenericutes | Mollicutes | AM275436_o | EF445272_f | EF445272_g | EF445272_f EF445272_g |
| Bacteria | Firmicutes | Erysipelotrichi | Erysipelotrichales | Erysipelotrichaceae | AJ629069_g | Erysipelotrichaceae AJ629069_g |
| Bacteria | Firmicutes | Clostridia | Clostridiales | Mogibacterium_f | AM500802_g | Mogibacterium_f AM500802_g |
| Bacteria | Bacteroidetes | Bacteroidia | Bacteroidales | Porphyromonadaceae | Paludibacter | Porphyromonadaceae Paludibacter |
| Bacteria | Firmicutes | Clostridia | Clostridiales | Lachnospiraceae | GU174097_g | Lachnospiraceae GU174097_g |
| Bacteria | Firmicutes | Bacilli | Bacillales | Paenibacillaceae | Paenibacillus | Paenibacillaceae Paenibacillus |
| Bacteria | Bacteroidetes | Bacteroidia | Bacteroidales | S24-7_f | JX096203_g | S24-7_f JX096203_g |
| Bacteria | Proteobacteria | Alphaproteobacteria | Rickettsiales | Paracaedibacteraceae | AB198590_g | Paracaedibacteraceae AB198590_g |
| Bacteria | Proteobacteria | Deltaproteobacteria | Bradymonadales | AB185724_f | AB185724_g | AB185724_f AB185724_g |
| Bacteria | Firmicutes | Clostridia | Clostridiales | Christensenellaceae | HM124260_g | Christensenellaceae HM124260_g |
| Bacteria | Firmicutes | Bacilli | Lactobacillales | Streptococcaceae | Lactococcus | Streptococcaceae Lactococcus |
| Bacteria | Firmicutes | Clostridia | Clostridiales | Lachnospiraceae | JX094957_g | Lachnospiraceae JX094957_g |
| Bacteria | Firmicutes | Clostridia | Clostridiales | Ruminococcaceae | Eubacterium_g8 | Ruminococcaceae Eubacterium_g8 |
| Bacteria | Elusimicrobia | Elusimicrobia_c | Elusimicrobiales | Elusimicrobiaceae | Elusimicrobium | Elusimicrobiaceae Elusimicrobium |
| Bacteria | Bacteroidetes | Bacteroidia | Bacteroidales | Bacteroidaceae | EU465631_g | Bacteroidaceae EU465631_g |
| Bacteria | Proteobacteria | Betaproteobacteria | Burkholderiales | Sutterellaceae | Parasutterella | Sutterellaceae Parasutterella |
| Bacteria | Firmicutes | Negativicutes | Selenomonadales | Selenomonadaceae | Schwartzia | Selenomonadaceae Schwartzia |
| Bacteria | Firmicutes | Clostridia | Clostridiales | Lachnospiraceae | EU358717_g | Lachnospiraceae EU358717_g |
| Bacteria | Firmicutes | Clostridia | Clostridiales | Lachnospiraceae | KE159538_g | Lachnospiraceae KE159538_g |
| Bacteria | Proteobacteria | Gammaproteobacteria | Cardiobacteriales | Cardiobacteriaceae |  | Cardiobacteriaceae |
| Bacteria | Bacteroidetes | Bacteroidia | Bacteroidales | Porphyromonadaceae | Parabacteroides | Porphyromonadaceae Parabacteroides |
| Bacteria | Firmicutes | Clostridia | Clostridiales | Lachnospiraceae | JPZU_g | Lachnospiraceae JPZU_g |
| Bacteria | Firmicutes | Clostridia | Clostridiales | Ruminococcaceae | AF018558_g | Ruminococcaceae AF018558_g |
| Bacteria | Firmicutes | Clostridia | Clostridiales | Lachnospiraceae | DQ057459_g | Lachnospiraceae DQ057459_g |
| Bacteria | Proteobacteria | Epsilonproteobacteria | Campylobacterales | Helicobacteraceae | Helicobacter | Helicobacteraceae Helicobacter |
| Bacteria | Bacteroidetes | Bacteroidia | Bacteroidales | GU304534_f | EU462208_g | GU304534_f EU462208_g |
| Bacteria | Firmicutes | Clostridia | Clostridiales | Ruminococcaceae | Butyricicoccus | Ruminococcaceae Butyricicoccus |
| Bacteria | Firmicutes | Clostridia | Clostridiales | Ruminococcaceae | EU843993_g | Ruminococcaceae EU843993_g |
| Bacteria | Firmicutes | Clostridia | Clostridiales | Lachnospiraceae | EU728721_g | Lachnospiraceae EU728721_g |
| Bacteria | Spirochaetes | Spirochaetes_c | Spirochaetales | Leptospiraceae | GQ468580_g | Leptospiraceae GQ468580_g |
| Bacteria | Firmicutes | Erysipelotrichi | Erysipelotrichales | Erysipelotrichaceae | EU771685_g | Erysipelotrichaceae EU771685_g |
| Bacteria | Bacteroidetes | Bacteroidia | Bacteroidales | Prevotellaceae |  | Prevotellaceae |
| Bacteria | Firmicutes | Clostridia | Clostridiales | Lachnospiraceae | Howardella | Lachnospiraceae Howardella |
| Bacteria | Firmicutes | Negativicutes | Selenomonadales | Selenomonadaceae | EU463474_g | Selenomonadaceae EU463474_g |
| Bacteria | Firmicutes | Clostridia | Clostridiales | Mogibacterium_f | EU842499_g | Mogibacterium_f EU842499_g |
| Bacteria | Proteobacteria | Alphaproteobacteria | Rhodospirillales | Acetobacteraceae | Gluconobacter | Acetobacteraceae Gluconobacter |
| Bacteria | Firmicutes | Clostridia | Clostridiales | Lachnospiraceae | EU844681_g | Lachnospiraceae EU844681_g |
| Bacteria | Bacteroidetes | Bacteroidia | Bacteroidales | S24-7_f |  | S24-7_f |
| Bacteria | Firmicutes | Clostridia | Clostridiales | Ruminococcaceae | Caproiciproducens | Ruminococcaceae Caproiciproducens |
| Bacteria | Tenericutes | Mollicutes | AM275436_o | AM275436_f | EU381820_g | AM275436_f EU381820_g |
| Bacteria | Actinobacteria | Coriobacteriia | Coriobacteriales | Coriobacteriaceae | Olsenella | Coriobacteriaceae Olsenella |
| Bacteria | Bacteroidetes | Bacteroidia | Bacteroidales | Anaerocella_f | Anaerocella | Anaerocella_f Anaerocella |
| Bacteria | Synergistetes | Synergistia | Synergistales | Fretibacterium_f | Fretibacterium | Fretibacterium_f Fretibacterium |
| Bacteria | Tenericutes | Mollicutes | Acholeplasmatales | Acholeplasmataceae | HM630232_g | Acholeplasmataceae HM630232_g |
| Bacteria | Firmicutes | Erysipelotrichi | Erysipelotrichales | Erysipelotrichaceae | Coprobacillus | Erysipelotrichaceae Coprobacillus |
| Bacteria | Firmicutes | Clostridia | Clostridiales | Lachnospiraceae | Roseburia | Lachnospiraceae Roseburia |
| Bacteria | Firmicutes | Erysipelotrichi | Erysipelotrichales | Erysipelotrichaceae | HQ806051_g | Erysipelotrichaceae HQ806051_g |
| Bacteria | Bacteroidetes | Bacteroidia | Bacteroidales | EU845084_f | EU842575_g | EU845084_f EU842575_g |
| Bacteria | Firmicutes | Clostridia | Clostridiales | Lachnospiraceae | Dorea | Lachnospiraceae Dorea |
| Bacteria | Firmicutes | Clostridia | Clostridiales | Mogibacterium_f | Eubacterium_g11 | Mogibacterium_f Eubacterium_g11 |
| Bacteria | Firmicutes | Clostridia | Clostridiales | Mogibacterium_f | GU174072_g | Mogibacterium_f GU174072_g |
| Bacteria | Firmicutes | Clostridia | Clostridiales | Lachnospiraceae | DQ353911_g | Lachnospiraceae DQ353911_g |
| Bacteria | Firmicutes | Bacilli | Bacillales | Staphylococcaceae | Macrococcus | Staphylococcaceae Macrococcus |
| Bacteria | Firmicutes | Negativicutes | Selenomonadales | Selenomonadaceae | AB185620_g | Selenomonadaceae AB185620_g |
| Bacteria | Verrucomicrobia | Opitutae | GU305779_o | EU464362_f | EU464362_g | EU464362_f EU464362_g |
| Bacteria | Tenericutes | Mollicutes | Mycoplasmatales | Mycoplasmataceae_f1 | Mycoplasma_g19 | Mycoplasmataceae_f1 Mycoplasma_g19 |
| Bacteria | Firmicutes | Erysipelotrichi | Erysipelotrichales | Erysipelotrichaceae | EU475451_g | Erysipelotrichaceae EU475451_g |
| Bacteria | Firmicutes | Clostridia | Clostridiales |  |  | Clostridiales |
| Bacteria | Proteobacteria | Betaproteobacteria | Burkholderiales | Ralstonia_f | Ralstonia | Ralstonia_f Ralstonia |
| Bacteria | Proteobacteria | Deltaproteobacteria | Desulfobulbaceae_o | Desulfobulbaceae |  | Desulfobulbaceae |
| Bacteria | Firmicutes | Erysipelotrichi | Erysipelotrichales | Erysipelotrichaceae | Bulleidia | Erysipelotrichaceae Bulleidia |
| Bacteria | Firmicutes | Clostridia | Clostridiales | Lachnospiraceae | Pseudobutyrivibrio | Lachnospiraceae Pseudobutyrivibrio |
| Bacteria | Firmicutes | Clostridia | Clostridiales | Lachnospiraceae | Clostridium_g7 | Lachnospiraceae Clostridium_g7 |
| Bacteria | Firmicutes | Clostridia | Clostridiales | Lachnospiraceae | GU324367_g | Lachnospiraceae GU324367_g |
| Bacteria | Firmicutes | Clostridia | Clostridiales | Christensenellaceae | FJ848448_g | Christensenellaceae FJ848448_g |
| Bacteria | Firmicutes | Clostridia | Clostridiales | Mogibacterium_f | FJ879347_g | Mogibacterium_f FJ879347_g |
| Bacteria | Firmicutes | Erysipelotrichi | Erysipelotrichales | Erysipelotrichaceae | EU842778_g | Erysipelotrichaceae EU842778_g |
| Bacteria | Firmicutes | Clostridia | Clostridiales | Mogibacterium_f | Aminicella | Mogibacterium_f Aminicella |
| Bacteria | Firmicutes | Clostridia | Clostridiales | Ruminococcaceae | JX198636_g | Ruminococcaceae JX198636_g |
| Bacteria | EU844128_p | EU844128_c | EU844128_o | EU844128_f | EU844128_g | EU844128_f EU844128_g |
| Bacteria | Firmicutes | Clostridia | Clostridiales | Lachnospiraceae | EU381644_g | Lachnospiraceae EU381644_g |
| Bacteria | Bacteroidetes | Bacteroidia | Bacteroidales | EU845084_f | AY244968_g | EU845084_f AY244968_g |
| Bacteria | Firmicutes | Bacilli | Bacillales | Planococcaceae | Kurthia | Planococcaceae Kurthia |
| Bacteria | Firmicutes | Erysipelotrichi | Erysipelotrichales | Erysipelotrichaceae | EU794282_g | Erysipelotrichaceae EU794282_g |
| Bacteria | Proteobacteria | Alphaproteobacteria | Rhodospirillales | Acetobacteraceae |  | Acetobacteraceae |
| Bacteria | Firmicutes | Clostridia | Clostridiales | Lachnospiraceae | Butyrivibrio_g1 | Lachnospiraceae Butyrivibrio_g1 |
| Bacteria | Bacteroidetes | Bacteroidia | Bacteroidales | EU845084_f | EU843760_g | EU845084_f EU843760_g |
| Bacteria | Firmicutes | Clostridia | Clostridiales | Lachnospiraceae | Coprococcus_g1 | Lachnospiraceae Coprococcus_g1 |
| Bacteria | Firmicutes | Clostridia | Clostridiales | Ruminococcaceae | FJ880290_g | Ruminococcaceae FJ880290_g |
| Bacteria | Firmicutes | Clostridia | Clostridiales | Lachnospiraceae | LLKB_g | Lachnospiraceae LLKB_g |
| Bacteria | Proteobacteria | Alphaproteobacteria | Rhodospirillales | Rhodospirillaceae | AB270041_g | Rhodospirillaceae AB270041_g |
| Bacteria | Firmicutes | Clostridia | Clostridiales | Christensenellaceae |  | Christensenellaceae |
| Bacteria | Spirochaetes | Spirochaetes_c | Spirochaetales | GQ249604_f | GQ249604_g | GQ249604_f GQ249604_g |
| Bacteria | Bacteroidetes | Bacteroidia | Bacteroidales | Odoribacteraceae | Odoribacter | Odoribacteraceae Odoribacter |
| Bacteria | Firmicutes | Clostridia | Clostridiales | Ruminococcaceae | EF404788_g | Ruminococcaceae EF404788_g |
| Bacteria | Bacteroidetes | Bacteroidia | Bacteroidales | Prevotellaceae | HQ399802_g | Prevotellaceae HQ399802_g |
| Bacteria | Firmicutes | Clostridia | Clostridiales | Christensenellaceae | AY442821_g | Christensenellaceae AY442821_g |
| Bacteria | Proteobacteria | Alphaproteobacteria | Rickettsiales | FJ478621_f | JX096224_g | FJ478621_f JX096224_g |
| Bacteria | Firmicutes | Clostridia | Clostridiales | Ruminococcaceae | EU844100_g | Ruminococcaceae EU844100_g |
| Bacteria | Firmicutes | Clostridia | Clostridiales | Lachnospiraceae | DQ115988_g | Lachnospiraceae DQ115988_g |
| Bacteria | Firmicutes | Clostridia | Clostridiales | Mogibacterium_f | EU381776_g | Mogibacterium_f EU381776_g |
| Bacteria | Firmicutes | Clostridia | Clostridiales | EU234093_f | EU234093_g | EU234093_f EU234093_g |
| Bacteria | Bacteroidetes | Bacteroidia | Bacteroidales | Prevotellaceae | Paraprevotella | Prevotellaceae Paraprevotella |
| Bacteria | Actinobacteria | Actinobacteria_c | Corynebacteriales | Corynebacteriaceae | Corynebacterium | Corynebacteriaceae Corynebacterium |
| Bacteria | Bacteroidetes | Bacteroidia | Bacteroidales | Bacteroidaceae | EU475549_g | Bacteroidaceae EU475549_g |
| Bacteria | Actinobacteria | Coriobacteriia | Coriobacteriales | Coriobacteriaceae | Parvibacter | Coriobacteriaceae Parvibacter |
| Bacteria | Firmicutes | Clostridia | Clostridiales | Lachnospiraceae | AB559589_g | Lachnospiraceae AB559589_g |
| Bacteria | Elusimicrobia | Elusimicrobia_c | Endomicrobium_o | Endomicrobium_f | Endomicrobium | Endomicrobium_f Endomicrobium |
| Bacteria | Firmicutes | Clostridia | Clostridiales | Ruminococcaceae | EF602946_g | Ruminococcaceae EF602946_g |
| Bacteria | Bacteroidetes | Bacteroidia | Bacteroidales | Porphyromonadaceae | Tannerella | Porphyromonadaceae Tannerella |
| Bacteria | Spirochaetes | Spirochaetes_c | Spirochaetales | Spirochaetaceae | HM049880_g | Spirochaetaceae HM049880_g |
| Bacteria | Bacteroidetes | Bacteroidia | Bacteroidales | Porphyromonadaceae | EU475408_g | Porphyromonadaceae EU475408_g |
| Bacteria | Proteobacteria | Alphaproteobacteria | Rickettsiales | FJ478621_f | GU303008_g | FJ478621_f GU303008_g |
| Bacteria | Firmicutes | Clostridia | Clostridiales | Christensenellaceae | HM124151_g | Christensenellaceae HM124151_g |
| Bacteria | Chloroflexi | Anaerolineae | Anaerolinaeles | Anaerolinaceae | Flexilinea | Anaerolinaceae Flexilinea |
| Bacteria | Firmicutes | Erysipelotrichi | Erysipelotrichales | Erysipelotrichaceae | EU842803_g | Erysipelotrichaceae EU842803_g |
| Bacteria | Bacteroidetes | Flavobacteria | Flavobacteriales | Flavobacteriaceae | Chryseobacterium | Flavobacteriaceae Chryseobacterium |
| Bacteria | Actinobacteria | Coriobacteriia | Coriobacteriales | Coriobacteriaceae | AM277063_g | Coriobacteriaceae AM277063_g |
| Bacteria | Proteobacteria | Gammaproteobacteria | Pseudomonadales | Moraxellaceae | Acinetobacter | Moraxellaceae Acinetobacter |
| Bacteria | Firmicutes | Clostridia | Clostridiales | Lachnospiraceae | EU137610_g | Lachnospiraceae EU137610_g |
| Bacteria | Firmicutes | Bacilli | Lactobacillales | Carnobacteriaceae | Carnobacterium | Carnobacteriaceae Carnobacterium |
| Bacteria | Firmicutes | Clostridia | Clostridiales | EU234093_f | AY426448_g | EU234093_f AY426448_g |
| Bacteria | Lentisphaerae | Lentisphaeria | Victivallales | Victivallaceae | Victivallis | Victivallaceae Victivallis |
| Bacteria | Actinobacteria | Coriobacteriia | Coriobacteriales | Coriobacteriaceae | Atopobium | Coriobacteriaceae Atopobium |
| Bacteria | Firmicutes | Clostridia | Clostridiales | Ruminococcaceae | GQ175418_g | Ruminococcaceae GQ175418_g |
| Bacteria | Firmicutes | Bacilli | Lactobacillales | Leuconostocaceae | Weissella | Leuconostocaceae Weissella |
| Bacteria | Bacteroidetes | Sphingobacteriia | Sphingobacteriales | Sphingobacteriaceae | Sphingobacterium | Sphingobacteriaceae Sphingobacterium |
| Bacteria | Firmicutes | Clostridia | Clostridiales | Lachnospiraceae | Agathobacter | Lachnospiraceae Agathobacter |
| Bacteria | Firmicutes | Clostridia | Clostridiales | Lachnospiraceae | Clostridium_g12 | Lachnospiraceae Clostridium_g12 |
| Bacteria | Firmicutes | Clostridia | Clostridiales | Lachnospiraceae | AY305316_g | Lachnospiraceae AY305316_g |
| Bacteria | Tenericutes | Mollicutes | AM275436_o | EF445272_f | EU844239_g | EF445272_f EU844239_g |
| Bacteria | Firmicutes | Erysipelotrichi | Erysipelotrichales | Erysipelotrichaceae | EU844830_g | Erysipelotrichaceae EU844830_g |
| Bacteria | Firmicutes | Clostridia | Clostridiales | Lachnospiraceae | EU381914_g | Lachnospiraceae EU381914_g |
| Bacteria | Firmicutes | Erysipelotrichi | Erysipelotrichales | Erysipelotrichaceae | Kandleria | Erysipelotrichaceae Kandleria |
| Bacteria | Firmicutes | Erysipelotrichi | Erysipelotrichales | Erysipelotrichaceae | GQ871718_g | Erysipelotrichaceae GQ871718_g |
| Bacteria | Firmicutes | Clostridia | Clostridiales | Peptostreptococcaceae |  | Peptostreptococcaceae |
| Bacteria | Firmicutes | Clostridia | Clostridiales | Lachnospiraceae | Moryella | Lachnospiraceae Moryella |
| Bacteria | Bacteroidetes | Bacteroidia | Bacteroidales | EU845084_f | AB270144_g | EU845084_f AB270144_g |
| Bacteria | Firmicutes | Clostridia | Clostridiales | Peptostreptococcaceae | Paraclostridium | Peptostreptococcaceae Paraclostridium |
| Bacteria | Proteobacteria | Gammaproteobacteria | Aeromonadales | Succinivibrionaceae | Succinimonas | Succinivibrionaceae Succinimonas |
| Bacteria | Lentisphaerae | Lentisphaeria | Victivallales | AB185535_f | GU304204_g | AB185535_f GU304204_g |
| Bacteria | Firmicutes | Clostridia | Clostridiales | Ruminococcaceae | KE993550_g | Ruminococcaceae KE993550_g |
| Bacteria | Firmicutes | Clostridia | Clostridiales | Ruminococcaceae | Subdoligranulum | Ruminococcaceae Subdoligranulum |
| Bacteria | Firmicutes | Clostridia | Clostridiales | Mogibacterium_f | EF604613_g | Mogibacterium_f EF604613_g |
| Bacteria | Synergistetes | Synergistia | Synergistales | Jonquetella_f | EU382030_g | Jonquetella_f EU382030_g |
| Bacteria | Firmicutes | Clostridia | Clostridiales | Christensenellaceae | FJ848389_g | Christensenellaceae FJ848389_g |
| Bacteria | Firmicutes | Clostridia | Clostridiales | Ruminococcaceae | EU381487_g | Ruminococcaceae EU381487_g |
| Bacteria | Firmicutes | Clostridia | Clostridiales | Lachnospiraceae | GQ458237_g | Lachnospiraceae GQ458237_g |
| Bacteria | Firmicutes | Erysipelotrichi | Erysipelotrichales | Erysipelotrichaceae | AB009222_g | Erysipelotrichaceae AB009222_g |
| Bacteria | Bacteroidetes | Bacteroidia | Bacteroidales | S24-7_f | EU461555_g | S24-7_f EU461555_g |
| Bacteria | Verrucomicrobia | Opitutae | FN377789_o | FN377789_f | FN377789_g | FN377789_f FN377789_g |
| Bacteria | Firmicutes | Clostridia | Clostridiales | Lachnospiraceae | Eubacterium_g17 | Lachnospiraceae Eubacterium_g17 |
| Bacteria | Firmicutes | Clostridia | Clostridiales | Lachnospiraceae | AJ576336_g | Lachnospiraceae AJ576336_g |
| Bacteria | Planctomycetes | Planctomycetia | Planctomycetales | Planctomycetaceae | AB506368_g | Planctomycetaceae AB506368_g |
| Bacteria | Actinobacteria | Actinobacteria_c | Actinomycetales | Actinomycetaceae | Actinobaculum | Actinomycetaceae Actinobaculum |
| Bacteria | SR1 | CP006913_c | CP006913_o | CP006913_f |  | CP006913_f |
| Bacteria | Peregrinibacteria | Peribacteria | ASND_o | ASND_f | ASND_g | ASND_f ASND_g |
| Bacteria | Planctomycetes | Planctomycetia | Planctomycetales | Planctomycetaceae |  | Planctomycetaceae |
| Bacteria | Tenericutes | Mollicutes | AM275436_o | AM275436_f |  | AM275436_f |
| Bacteria | Bacteroidetes | Bacteroidia | Bacteroidales | EU845084_f | EU259392_g | EU845084_f EU259392_g |
| Bacteria | Actinobacteria | Coriobacteriia | Coriobacteriales | Coriobacteriaceae | AX003092_g | Coriobacteriaceae AX003092_g |
| Bacteria | Proteobacteria | Deltaproteobacteria | Desulfobulbaceae_o | Desulfobulbaceae | AY771958_g | Desulfobulbaceae AY771958_g |
| Bacteria | Bacteroidetes | Flavobacteria | Flavobacteriales |  |  | Flavobacteriales |
| Bacteria | Actinobacteria | Coriobacteriia | Coriobacteriales | Coriobacteriaceae | EU504948_g | Coriobacteriaceae EU504948_g |
| Bacteria | Actinobacteria | Actinobacteria_c | Streptomycetales | Streptomycetaceae | Streptomyces | Streptomycetaceae Streptomyces |
| Bacteria | Bacteroidetes | Bacteroidia | Bacteroidales | RF16_f | EU459465_g | RF16_f EU459465_g |
| Bacteria | Verrucomicrobia | Opitutae | FN377789_o | AY571501_f | EU850497_g | AY571501_f EU850497_g |
| Bacteria | Firmicutes | Clostridia | Clostridiales | Lachnospiraceae | DQ394641_g | Lachnospiraceae DQ394641_g |
| Bacteria | Firmicutes | Bacilli | Lactobacillales | Carnobacteriaceae | Desemzia | Carnobacteriaceae Desemzia |
| Bacteria | Bacteroidetes | Bacteroidia | Bacteroidales | EU845084_f | KC163097_g | EU845084_f KC163097_g |
| Bacteria | Bacteroidetes | __ | __ |  |  | Bacteroidetes |
| Bacteria | Firmicutes | Clostridia | Clostridiales | Ruminococcaceae | Acetanaerobacterium | Ruminococcaceae Acetanaerobacterium |
| Bacteria | Firmicutes | Clostridia | Clostridiales | Christensenellaceae | HM124244_g | Christensenellaceae HM124244_g |
| Bacteria | Firmicutes | Clostridia | Clostridiales | Lachnospiraceae | Clostridium_g11 | Lachnospiraceae Clostridium_g11 |
| Bacteria | Firmicutes | Clostridia | Clostridiales | Lachnospiraceae | JNIN_g | Lachnospiraceae JNIN_g |
| Bacteria | Firmicutes | Clostridia | Clostridiales | Lachnospiraceae | Lactonifactor | Lachnospiraceae Lactonifactor |
| Bacteria | Tenericutes | Mollicutes | GU196243_o | FJ367735_f | FJ367735_g | FJ367735_f FJ367735_g |
| Bacteria | Firmicutes | Clostridia | Clostridiales | Ruminococcaceae | FJ951854_g | Ruminococcaceae FJ951854_g |
| Bacteria | Proteobacteria | Alphaproteobacteria | Rickettsiales | AY571491_f | AJ428412_g | AY571491_f AJ428412_g |
| Bacteria | Firmicutes | Clostridia | Clostridiales | Ruminococcaceae | DQ777889_g | Ruminococcaceae DQ777889_g |
| Bacteria | Lentisphaerae | Lentisphaeria | Victivallales | Victivallaceae | GQ134404_g | Victivallaceae GQ134404_g |
| Bacteria | Proteobacteria | Oligoflexia | Oligoflexales | Oligoflexaceae |  | Oligoflexaceae |
| Bacteria | Firmicutes | Clostridia | Clostridiales | Ruminococcaceae | AY854276_g | Ruminococcaceae AY854276_g |
| Bacteria | Firmicutes | Clostridia | Clostridiales | Ruminococcaceae | EU844075_g | Ruminococcaceae EU844075_g |
| Bacteria | Firmicutes | Clostridia | Clostridiales | Ruminococcaceae | HM124225_g | Ruminococcaceae HM124225_g |
| Bacteria | Firmicutes | Clostridia | Clostridiales | Ruminococcaceae | EU794101_g | Ruminococcaceae EU794101_g |
| Bacteria | Bacteroidetes | Bacteroidia | Bacteroidales | AM982614_f | AM982614_g | AM982614_f AM982614_g |
| Bacteria | Bacteroidetes | Bacteroidia | Bacteroidales | EU845084_f |  | EU845084_f |
| Bacteria | Spirochaetes | Spirochaetes_c | Spirochaetales | Spirochaetaceae | AJ431240_g | Spirochaetaceae AJ431240_g |
| Bacteria | Firmicutes | Clostridia | Clostridiales | Ruminococcaceae | Clostridium_g18 | Ruminococcaceae Clostridium_g18 |
| Bacteria | Firmicutes | Clostridia | Clostridiales | Lachnospiraceae | FJ825526_g | Lachnospiraceae FJ825526_g |
| Bacteria | Firmicutes | Clostridia | Clostridiales | Peptostreptococcaceae | Terrisporobacter | Peptostreptococcaceae Terrisporobacter |
| Bacteria | Firmicutes | Bacilli | Lactobacillales | Aerococcaceae | Aerococcus | Aerococcaceae Aerococcus |
| Bacteria | Bacteroidetes | Bacteroidia | Bacteroidales | Porphyromonadaceae | Coprobacter | Porphyromonadaceae Coprobacter |
| Bacteria | Firmicutes | Clostridia | Clostridiales | Lachnospiraceae | AJ518873_g | Lachnospiraceae AJ518873_g |
| Bacteria | Firmicutes | Clostridia | Clostridiales | Ruminococcaceae | EF400272_g | Ruminococcaceae EF400272_g |
| Bacteria | Firmicutes | Erysipelotrichi | Erysipelotrichales | Erysipelotrichaceae | Eubacterium_g1 | Erysipelotrichaceae Eubacterium_g1 |
| Bacteria | Lentisphaerae | Lentisphaeria | Victivallales | Victivallaceae | EU471634_g | Victivallaceae EU471634_g |
| Bacteria | Firmicutes | Clostridia | Clostridiales | Lachnospiraceae | EU845632_g | Lachnospiraceae EU845632_g |
| Bacteria | Bacteroidetes | Bacteroidia | Bacteroidales | RF16_f | EU471633_g | RF16_f EU471633_g |
| Bacteria | Bacteroidetes | Flavobacteria | Flavobacteriales | Flavobacteriaceae | EU779437_g | Flavobacteriaceae EU779437_g |
| Bacteria | Bacteroidetes | Flavobacteria | Flavobacteriales | Flavobacteriaceae |  | Flavobacteriaceae |
| Bacteria | Bacteroidetes | Bacteroidia | Bacteroidales | Prevotellaceae | GQ451241_g | Prevotellaceae GQ451241_g |
| Bacteria | Firmicutes | Erysipelotrichi | Erysipelotrichales | Erysipelotrichaceae | DQ456144_g | Erysipelotrichaceae DQ456144_g |
| Bacteria | Firmicutes | Clostridia | Clostridiales | Christensenellaceae | GQ448104_g | Christensenellaceae GQ448104_g |
| Bacteria | Proteobacteria | Deltaproteobacteria | Desulfuromonadales | Desulfuromonadaceae |  | Desulfuromonadaceae |
| Bacteria | Bacteroidetes | Bacteroidia | Bacteroidales | JF747991_f | FJ437753_g | JF747991_f FJ437753_g |
| Bacteria | Firmicutes | Clostridia | Clostridiales | Ruminococcaceae | EU772127_g | Ruminococcaceae EU772127_g |
| Bacteria | Firmicutes | Clostridia | Clostridiales | Lachnospiraceae | AB185516_g | Lachnospiraceae AB185516_g |
| Bacteria | SR1 | CP006913_c | CP006913_o | CP006913_f | JN429833_g | CP006913_f JN429833_g |
| Bacteria | Tenericutes | Mollicutes | GU196243_o | FJ367735_f | GQ451208_g | FJ367735_f GQ451208_g |
| Bacteria | Firmicutes | Clostridia | Clostridiales | Christensenellaceae | GQ138168_g | Christensenellaceae GQ138168_g |
| Bacteria | Tenericutes | Mollicutes | GU196243_o | FJ367735_f | JX096375_g | FJ367735_f JX096375_g |
| Bacteria | Bacteroidetes | Bacteroidia | Bacteroidales | GU304534_f | EU474499_g | GU304534_f EU474499_g |
| Bacteria | Bacteroidetes | Bacteroidia | Bacteroidales | BS11_f | EU407205_g | BS11_f EU407205_g |
| Bacteria | Firmicutes | Erysipelotrichi | Erysipelotrichales | Erysipelotrichaceae | Catenisphaera | Erysipelotrichaceae Catenisphaera |
| Bacteria | Bacteroidetes | Bacteroidia | Bacteroidales | S24-7_f | EU791023_g | S24-7_f EU791023_g |
| Bacteria | Firmicutes | Clostridia | Clostridiales | Ruminococcaceae | EU842424_g | Ruminococcaceae EU842424_g |
| Bacteria | Proteobacteria | Alphaproteobacteria | Rhodospirillales | Rhodospirillaceae | JN713244_g | Rhodospirillaceae JN713244_g |
| Bacteria | Tenericutes | Mollicutes | Acholeplasmatales | Acholeplasmataceae | AB598274_g | Acholeplasmataceae AB598274_g |
| Bacteria | Firmicutes | Bacilli | Lactobacillales | Leuconostocaceae | Leuconostoc | Leuconostocaceae Leuconostoc |
| Bacteria | Proteobacteria | Deltaproteobacteria | Desulfovibrionales | Desulfovibrionaceae |  | Desulfovibrionaceae |
| Bacteria | Firmicutes | Clostridia | Clostridiales | Christensenellaceae | JN162687_g | Christensenellaceae JN162687_g |
| Bacteria | Bacteroidetes | Bacteroidia | Bacteroidales | Porphyromonadaceae | HQ183936_g | Porphyromonadaceae HQ183936_g |
| Bacteria | Firmicutes | Clostridia | Clostridiales | Lachnospiraceae | EU842423_g | Lachnospiraceae EU842423_g |
| Bacteria | Firmicutes | Clostridia | Clostridiales | Ruminococcaceae | FJ951890_g | Ruminococcaceae FJ951890_g |
| Bacteria | Spirochaetes | Spirochaetes_c | Spirochaetales | Spirochaetaceae | Sphaerochaeta | Spirochaetaceae Sphaerochaeta |
| Bacteria | Proteobacteria | Betaproteobacteria | Burkholderiales | Sutterellaceae | Sutterella | Sutterellaceae Sutterella |
| Bacteria | Firmicutes | Clostridia | Clostridiales | Ruminococcaceae | HM124015_g | Ruminococcaceae HM124015_g |
| Bacteria | Bacteroidetes | Bacteroidia | Bacteroidales | EU845084_f | AB270099_g | EU845084_f AB270099_g |
| Bacteria | Firmicutes | Clostridia | Clostridiales | Lachnospiraceae | DQ904798_g | Lachnospiraceae DQ904798_g |
| Bacteria | Firmicutes | Clostridia | Clostridiales | Mogibacterium_f | EU460979_g | Mogibacterium_f EU460979_g |
| Bacteria | Verrucomicrobia | Opitutae | GU305779_o | GU305779_f | EU469726_g | GU305779_f EU469726_g |
| Bacteria | Bacteroidetes | Bacteroidia | Bacteroidales | EU845084_f | EU794254_g | EU845084_f EU794254_g |
| Bacteria | Firmicutes | Clostridia | Clostridiales | Ruminococcaceae | AY858456_g | Ruminococcaceae AY858456_g |
| Bacteria | Firmicutes | Negativicutes | Selenomonadales | Selenomonadaceae |  | Selenomonadaceae |
| Bacteria | Tenericutes | Mollicutes | Acholeplasmatales | Acholeplasmataceae |  | Acholeplasmataceae |
| Bacteria | Bacteroidetes | Bacteroidia | Bacteroidales | Porphyromonadaceae | EU471619_g | Porphyromonadaceae EU471619_g |
| Bacteria | Firmicutes | Clostridia | Clostridiales | Mogibacterium_f | AY134903_g | Mogibacterium_f AY134903_g |
| Bacteria | Lentisphaerae | Lentisphaeria | Victivallales | Victivallaceae |  | Victivallaceae |
| Bacteria | Proteobacteria | Betaproteobacteria | Rhodocyclales | Rhodocyclaceae | Azoarcus | Rhodocyclaceae Azoarcus |
| Bacteria | Bacteroidetes | Bacteroidia | Bacteroidales | Porphyromonadaceae | EU794128_g | Porphyromonadaceae EU794128_g |
| Bacteria | Peregrinibacteria | Peribacteria | ASND_o | ASND_f |  | ASND_f |
| Bacteria | Actinobacteria | Actinobacteria_c | Micrococcales | Micrococcaceae | Arthrobacter | Micrococcaceae Arthrobacter |
| Bacteria | Firmicutes | Clostridia | Clostridiales | Christensenellaceae | GQ422712_g | Christensenellaceae GQ422712_g |
| Bacteria | Firmicutes | Clostridia | Clostridiales | Eubacteriaceae | Eubacterium | Eubacteriaceae Eubacterium |
| Bacteria | Proteobacteria | Deltaproteobacteria | Desulfovibrionales | Desulfovibrionaceae | Desulfovibrio_g4 | Desulfovibrionaceae Desulfovibrio_g4 |
| Bacteria | Firmicutes | Clostridia | Clostridiales | Ruminococcaceae | EU791177_g | Ruminococcaceae EU791177_g |
| Bacteria | Firmicutes | Clostridia | Clostridiales | Ruminococcaceae | EU843612_g | Ruminococcaceae EU843612_g |
| Bacteria | Proteobacteria | Deltaproteobacteria | Bdellovibrionales | CU925466_f |  | CU925466_f |
| Bacteria | Tenericutes | Mollicutes | Mycoplasmatales | Mycoplasmataceae_f1 |  | Mycoplasmataceae_f1 |
| Bacteria | Bacteroidetes | Bacteroidia | Bacteroidales | S24-7_f | EF100102_g | S24-7_f EF100102_g |
| Bacteria | Firmicutes | Clostridia | Clostridiales | Lachnospiraceae | GU324393_g | Lachnospiraceae GU324393_g |
| Bacteria | Firmicutes | Erysipelotrichi | Erysipelotrichales | Erysipelotrichaceae | EU794124_g | Erysipelotrichaceae EU794124_g |
| Bacteria | Proteobacteria | Deltaproteobacteria | Desulfovibrionales | Desulfovibrionaceae | Bilophila | Desulfovibrionaceae Bilophila |
| Bacteria | Proteobacteria | Betaproteobacteria | Nitrosomonadales | Nitrosomonadaceae | Nitrosomonas | Nitrosomonadaceae Nitrosomonas |
| Bacteria | Firmicutes | Clostridia | Clostridiales | Christensenellaceae | JX095379_g | Christensenellaceae JX095379_g |
| Bacteria | Proteobacteria | Alphaproteobacteria | Rhodospirillales | Rhodospirillaceae | EU381783_g | Rhodospirillaceae EU381783_g |
| Bacteria | Proteobacteria | Gammaproteobacteria | Legionellales | Coxiellaceae |  | Coxiellaceae |
| Bacteria | Bacteroidetes | Bacteroidia | Bacteroidales | JF747991_f | JF747991_g | JF747991_f JF747991_g |
| Bacteria | Firmicutes | Erysipelotrichi | Erysipelotrichales | Erysipelotrichaceae | FJ681535_g | Erysipelotrichaceae FJ681535_g |
| Bacteria | Firmicutes | Clostridia | Clostridiales | Lachnospiraceae | DQ394608_g | Lachnospiraceae DQ394608_g |
| Bacteria | Firmicutes | Clostridia | Clostridiales | Ruminococcaceae | EF436360_g | Ruminococcaceae EF436360_g |
| Bacteria | Bacteroidetes | Bacteroidia | Bacteroidales | GU454901_f | GU454901_g | GU454901_f GU454901_g |
| Bacteria | Actinobacteria | Coriobacteriia | Coriobacteriales | Coriobacteriaceae | AM278923_g | Coriobacteriaceae AM278923_g |
| Bacteria | Actinobacteria | Coriobacteriia | Coriobacteriales | Coriobacteriaceae | EU471836_g | Coriobacteriaceae EU471836_g |

**Table S6.** Taxonomic string of all genera in the Clostridia specific analysis

| Kingdom | Phylum | Class | Order | Family | Genus | Label |
| --- | --- | --- | --- | --- | --- | --- |
| Bacteria | Firmicutes | Clostridia | Clostridiales | Lachnospiraceae |  | Lachnospiraceae |
| Bacteria | Firmicutes | Clostridia | Clostridiales | Peptostreptococcaceae | Romboutsia | Peptostreptococcaceae Romboutsia |
| Bacteria | Firmicutes | Clostridia | Clostridiales | Peptostreptococcaceae |  | Peptostreptococcaceae |
| Bacteria | Firmicutes | Clostridia | Clostridiales | Christensenellaceae | AB239481_g | Christensenellaceae AB239481_g |
| Bacteria | Firmicutes | Clostridia | Clostridiales | Lachnospiraceae | Catonella | Lachnospiraceae Catonella |
| Bacteria | Firmicutes | Clostridia | Clostridiales | Mogibacterium_f | EU844817_g | Mogibacterium_f EU844817_g |
| Bacteria | Firmicutes | Clostridia | Clostridiales | Lachnospiraceae | Blautia | Lachnospiraceae Blautia |
| Bacteria | Firmicutes | Clostridia | Clostridiales | Clostridiaceae | Clostridium | Clostridiaceae Clostridium |
| Bacteria | Firmicutes | Clostridia | Clostridiales | Lachnospiraceae | FJ880395_g | Lachnospiraceae FJ880395_g |
| Bacteria | Firmicutes | Clostridia | Clostridiales | Lachnospiraceae | Butyrivibrio | Lachnospiraceae Butyrivibrio |
| Bacteria | Firmicutes | Clostridia | Clostridiales | Lachnospiraceae | Syntrophococcus | Lachnospiraceae Syntrophococcus |
| Bacteria | Firmicutes | Clostridia | Clostridiales | Lachnospiraceae | Eubacterium_g7 | Lachnospiraceae Eubacterium_g7 |
| Bacteria | Firmicutes | Clostridia | Clostridiales | Mogibacterium_f | EF436322_g | Mogibacterium_f EF436322_g |
| Bacteria | Firmicutes | Clostridia | Clostridiales | Christensenellaceae | GU302849_g | Christensenellaceae GU302849_g |
| Bacteria | Firmicutes | Clostridia | Clostridiales | Mogibacterium_f |  | Mogibacterium_f |
| Bacteria | Firmicutes | Clostridia | Clostridiales | Christensenellaceae | HQ716403_g | Christensenellaceae HQ716403_g |
| Bacteria | Firmicutes | Clostridia | Clostridiales | Mogibacterium_f | EU842499_g | Mogibacterium_f EU842499_g |
| Bacteria | Firmicutes | Clostridia | Clostridiales | Mogibacterium_f | Mogibacterium | Mogibacterium_f Mogibacterium |
| Bacteria | Firmicutes | Clostridia | Clostridiales | Mogibacterium_f | AM500802_g | Mogibacterium_f AM500802_g |
| Bacteria | Firmicutes | Clostridia | Clostridiales | Lachnospiraceae | Coprococcus_g2 | Lachnospiraceae Coprococcus_g2 |
| Bacteria | Firmicutes | Clostridia | Clostridiales | Lachnospiraceae | KE159538_g | Lachnospiraceae KE159538_g |
| Bacteria | Firmicutes | Clostridia | Clostridiales | Lachnospiraceae | Lachnospira | Lachnospiraceae Lachnospira |
| Bacteria | Firmicutes | Clostridia | Clostridiales | Lachnospiraceae | GU174103_g | Lachnospiraceae GU174103_g |
| Bacteria | Firmicutes | Clostridia | Clostridiales | Lachnospiraceae | GU174097_g | Lachnospiraceae GU174097_g |
| Bacteria | Firmicutes | Clostridia | Clostridiales | Lachnospiraceae | EU728721_g | Lachnospiraceae EU728721_g |
| Bacteria | Firmicutes | Clostridia | Clostridiales | Lachnospiraceae | Eubacterium_g4 | Lachnospiraceae Eubacterium_g4 |
| Bacteria | Firmicutes | Clostridia | Clostridiales | Lachnospiraceae | Eubacterium_g17 | Lachnospiraceae Eubacterium_g17 |
| Bacteria | Firmicutes | Clostridia | Clostridiales | Lachnospiraceae | EU842486_g | Lachnospiraceae EU842486_g |
| Bacteria | Firmicutes | Clostridia | Clostridiales | Mogibacterium_f | Aminicella | Mogibacterium_f Aminicella |
| Bacteria | Firmicutes | Clostridia | Clostridiales | Lachnospiraceae | EU358717_g | Lachnospiraceae EU358717_g |
| Bacteria | Firmicutes | Clostridia | Clostridiales | Lachnospiraceae | Oribacterium | Lachnospiraceae Oribacterium |
| Bacteria | Firmicutes | Clostridia | Clostridiales | Mogibacterium_f | EU844456_g | Mogibacterium_f EU844456_g |
| Bacteria | Firmicutes | Clostridia | Clostridiales | Christensenellaceae | Christensenella | Christensenellaceae Christensenella |
| Bacteria | Firmicutes | Clostridia | Clostridiales | Lachnospiraceae | Eubacterium_g5 | Lachnospiraceae Eubacterium_g5 |
| Bacteria | Firmicutes | Clostridia | Clostridiales | Lachnospiraceae | Roseburia | Lachnospiraceae Roseburia |
| Bacteria | Firmicutes | Clostridia | Clostridiales | Lachnospiraceae | Butyrivibrio_g1 | Lachnospiraceae Butyrivibrio_g1 |
| Bacteria | Firmicutes | Clostridia | Clostridiales | Lachnospiraceae | EU844681_g | Lachnospiraceae EU844681_g |
| Bacteria | Firmicutes | Clostridia | Clostridiales | Lachnospiraceae | EU137610_g | Lachnospiraceae EU137610_g |
| Bacteria | Firmicutes | Clostridia | Clostridiales | Lachnospiraceae | Moryella | Lachnospiraceae Moryella |
| Bacteria | Firmicutes | Clostridia | Clostridiales | Lachnospiraceae | Pseudobutyrivibrio | Lachnospiraceae Pseudobutyrivibrio |
| Bacteria | Firmicutes | Clostridia | Clostridiales | Lachnospiraceae | EU381914_g | Lachnospiraceae EU381914_g |
| Bacteria | Firmicutes | Clostridia | Clostridiales | Mogibacterium_f | GU174072_g | Mogibacterium_f GU174072_g |
| Bacteria | Firmicutes | Clostridia | Clostridiales | Ruminococcaceae | Pseudoflavonifractor | Ruminococcaceae Pseudoflavonifractor |
| Bacteria | Firmicutes | Clostridia | Clostridiales | Mogibacterium_f | EU381776_g | Mogibacterium_f EU381776_g |
| Bacteria | Firmicutes | Clostridia | Clostridiales | Lachnospiraceae | Howardella | Lachnospiraceae Howardella |
| Bacteria | Firmicutes | Clostridia | Clostridiales | Lachnospiraceae | Clostridium_g7 | Lachnospiraceae Clostridium_g7 |
| Bacteria | Firmicutes | Clostridia | Clostridiales | Lachnospiraceae | Clostridium_g11 | Lachnospiraceae Clostridium_g11 |
| Bacteria | Firmicutes | Clostridia | Clostridiales | Lachnospiraceae | EU381644_g | Lachnospiraceae EU381644_g |
| Bacteria | Firmicutes | Clostridia | Clostridiales | Ruminococcaceae | JN713389_g | Ruminococcaceae JN713389_g |
| Bacteria | Firmicutes | Clostridia | Clostridiales | Mogibacterium_f | FJ879347_g | Mogibacterium_f FJ879347_g |
| Bacteria | Firmicutes | Clostridia | Clostridiales | EU234093_f |  | EU234093_f |
| Bacteria | Firmicutes | Clostridia | Clostridiales | EU234093_f | EU234093_g | EU234093_f EU234093_g |
| Bacteria | Firmicutes | Clostridia | Clostridiales | Lachnospiraceae | Lachnobacterium | Lachnospiraceae Lachnobacterium |
| Bacteria | Firmicutes | Clostridia | Clostridiales | Lachnospiraceae | AB185516_g | Lachnospiraceae AB185516_g |
| Bacteria | Firmicutes | Clostridia | Clostridiales | Lachnospiraceae | Agathobacter | Lachnospiraceae Agathobacter |
| Bacteria | Firmicutes | Clostridia | Clostridiales | Lachnospiraceae | DQ115988_g | Lachnospiraceae DQ115988_g |
| Bacteria | Firmicutes | Clostridia | Clostridiales | Peptostreptococcaceae | Paraclostridium | Peptostreptococcaceae Paraclostridium |
| Bacteria | Firmicutes | Clostridia | Clostridiales | Lachnospiraceae | FJ680468_g | Lachnospiraceae FJ680468_g |
| Bacteria | Firmicutes | Clostridia | Clostridiales | Lachnospiraceae | JX094957_g | Lachnospiraceae JX094957_g |
| Bacteria | Firmicutes | Clostridia | Clostridiales | Mogibacterium_f | Anaerovorax | Mogibacterium_f Anaerovorax |
| Bacteria | Firmicutes | Clostridia | Clostridiales | Christensenellaceae | HM124260_g | Christensenellaceae HM124260_g |
| Bacteria | Firmicutes | Clostridia | Clostridiales | Christensenellaceae | AY442821_g | Christensenellaceae AY442821_g |
| Bacteria | Firmicutes | Clostridia | Clostridiales | Lachnospiraceae | JPZU_g | Lachnospiraceae JPZU_g |
| Bacteria | Firmicutes | Clostridia | Clostridiales | Lachnospiraceae | AY305316_g | Lachnospiraceae AY305316_g |
| Bacteria | Firmicutes | Clostridia | Clostridiales | Lachnospiraceae | Lactonifactor | Lachnospiraceae Lactonifactor |
| Bacteria | Firmicutes | Clostridia | Clostridiales | EU234093_f | AY426448_g | EU234093_f AY426448_g |
| Bacteria | Firmicutes | Clostridia | Clostridiales | Lachnospiraceae | JNIN_g | Lachnospiraceae JNIN_g |
| Bacteria | Firmicutes | Clostridia | Clostridiales | Peptostreptococcaceae | Terrisporobacter | Peptostreptococcaceae Terrisporobacter |
| Bacteria | Firmicutes | Clostridia | Clostridiales | Lachnospiraceae | EU845632_g | Lachnospiraceae EU845632_g |
| Bacteria | Firmicutes | Clostridia | Clostridiales | Lachnospiraceae | Coprococcus_g1 | Lachnospiraceae Coprococcus_g1 |
| Bacteria | Firmicutes | Clostridia | Clostridiales | Lachnospiraceae | AJ576336_g | Lachnospiraceae AJ576336_g |
| Bacteria | Firmicutes | Clostridia | Clostridiales | Christensenellaceae | JN162687_g | Christensenellaceae JN162687_g |
| Bacteria | Firmicutes | Clostridia | Clostridiales | Lachnospiraceae | AB506319_g | Lachnospiraceae AB506319_g |
| Bacteria | Firmicutes | Clostridia | Clostridiales | Lachnospiraceae | GU324367_g | Lachnospiraceae GU324367_g |
| Bacteria | Firmicutes | Clostridia | Clostridiales | Ruminococcaceae | Ruminococcus | Ruminococcaceae Ruminococcus |
| Bacteria | Firmicutes | Clostridia | Clostridiales | Christensenellaceae | FJ848448_g | Christensenellaceae FJ848448_g |
| Bacteria | Firmicutes | Clostridia | Clostridiales | Lachnospiraceae | JX096039_g | Lachnospiraceae JX096039_g |
| Bacteria | Firmicutes | Clostridia | Clostridiales | Ruminococcaceae |  | Ruminococcaceae |
| Bacteria | Firmicutes | Clostridia | Clostridiales | Lachnospiraceae | Clostridium_g8 | Lachnospiraceae Clostridium_g8 |
| Bacteria | Firmicutes | Clostridia | Clostridiales | Christensenellaceae | HM124244_g | Christensenellaceae HM124244_g |
| Bacteria | Firmicutes | Clostridia | Clostridiales | Christensenellaceae |  | Christensenellaceae |
| Bacteria | Firmicutes | Clostridia | Clostridiales |  |  | Clostridiales |
| Bacteria | Firmicutes | Clostridia | Clostridiales | Christensenellaceae | JX575947_g | Christensenellaceae JX575947_g |
| Bacteria | Firmicutes | Clostridia | Clostridiales | Lachnospiraceae | Eisenbergiella | Lachnospiraceae Eisenbergiella |
| Bacteria | Firmicutes | Clostridia | Clostridiales | Ruminococcaceae | AB009176_g | Ruminococcaceae AB009176_g |
| Bacteria | Firmicutes | Clostridia | Clostridiales | Lachnospiraceae | GQ458237_g | Lachnospiraceae GQ458237_g |
| Bacteria | Firmicutes | Clostridia | Clostridiales | Lachnospiraceae | FJ825526_g | Lachnospiraceae FJ825526_g |
| Bacteria | Firmicutes | Clostridia | Clostridiales | Christensenellaceae | U81670_g | Christensenellaceae U81670_g |
| Bacteria | Firmicutes | Clostridia | Clostridiales | Peptostreptococcaceae | Asaccharospora | Peptostreptococcaceae Asaccharospora |
| Bacteria | Firmicutes | Clostridia | Clostridiales | Ruminococcaceae | Sporobacter | Ruminococcaceae Sporobacter |
| Bacteria | Firmicutes | Clostridia | Clostridiales | Ruminococcaceae | Oscillibacter | Ruminococcaceae Oscillibacter |
| Bacteria | Firmicutes | Clostridia | Clostridiales | Lachnospiraceae | EU843171_g | Lachnospiraceae EU843171_g |
| Bacteria | Firmicutes | Clostridia | Clostridiales | Christensenellaceae | GQ422712_g | Christensenellaceae GQ422712_g |
| Bacteria | Firmicutes | Clostridia | Clostridiales | Lachnospiraceae | GU324408_g | Lachnospiraceae GU324408_g |
| Bacteria | Firmicutes | Clostridia | Clostridiales | Lachnospiraceae | AB559589_g | Lachnospiraceae AB559589_g |
| Bacteria | Firmicutes | Clostridia | Clostridiales | EU842383_f | EU842383_g | EU842383_f EU842383_g |
| Bacteria | Firmicutes | Clostridia | Clostridiales | Mogibacterium_f | AM982556_g | Mogibacterium_f AM982556_g |
| Bacteria | Firmicutes | Clostridia | Clostridiales | Christensenellaceae | HM124151_g | Christensenellaceae HM124151_g |
| Bacteria | Firmicutes | Clostridia | Clostridiales | Mogibacterium_f | EU460979_g | Mogibacterium_f EU460979_g |
| Bacteria | Firmicutes | Clostridia | Clostridiales | Lachnospiraceae | DQ394641_g | Lachnospiraceae DQ394641_g |
| Bacteria | Firmicutes | Clostridia | Clostridiales | Lachnospiraceae | EU845038_g | Lachnospiraceae EU845038_g |
| Bacteria | Firmicutes | Clostridia | Clostridiales | Christensenellaceae | GQ448104_g | Christensenellaceae GQ448104_g |
| Bacteria | Firmicutes | Clostridia | Clostridiales | Lachnospiraceae | Eubacterium_g21 | Lachnospiraceae Eubacterium_g21 |
| Bacteria | Firmicutes | Clostridia | Clostridiales | Peptococcaceae | EU622770_g | Peptococcaceae EU622770_g |
| Bacteria | Firmicutes | Clostridia | Clostridiales | Ruminococcaceae | AY854276_g | Ruminococcaceae AY854276_g |
| Bacteria | Firmicutes | Clostridia | Clostridiales | Ruminococcaceae | AF018558_g | Ruminococcaceae AF018558_g |
| Bacteria | Firmicutes | Clostridia | Clostridiales | Christensenellaceae | FJ848389_g | Christensenellaceae FJ848389_g |
| Bacteria | Firmicutes | Clostridia | Clostridiales | Lachnospiraceae | Cellulosilyticum | Lachnospiraceae Cellulosilyticum |
| Bacteria | Firmicutes | Clostridia | Clostridiales | Ruminococcaceae | Ruminococcus_g2 | Ruminococcaceae Ruminococcus_g2 |
| Bacteria | Firmicutes | Clostridia | Clostridiales | Lachnospiraceae | LLKB_g | Lachnospiraceae LLKB_g |
| Bacteria | Firmicutes | Clostridia | Clostridiales | Eubacteriaceae | Eubacterium | Eubacteriaceae Eubacterium |
| Bacteria | Firmicutes | Clostridia | Clostridiales | Eubacteriaceae | DQ353937_g | Eubacteriaceae DQ353937_g |
| Bacteria | Firmicutes | Clostridia | Clostridiales | Christensenellaceae | FJ374208_g | Christensenellaceae FJ374208_g |
| Bacteria | Firmicutes | Clostridia | Clostridiales | Ruminococcaceae | EU844075_g | Ruminococcaceae EU844075_g |
| Bacteria | Firmicutes | Clostridia | Clostridiales | Ruminococcaceae | EU842424_g | Ruminococcaceae EU842424_g |
| Bacteria | Firmicutes | Clostridia | Clostridiales | Ruminococcaceae | GQ175418_g | Ruminococcaceae GQ175418_g |
| Bacteria | Firmicutes | Clostridia | Clostridiales | Christensenellaceae | GQ451249_g | Christensenellaceae GQ451249_g |
| Bacteria | Firmicutes | Clostridia | Clostridiales | Ruminococcaceae | JX198636_g | Ruminococcaceae JX198636_g |
| Bacteria | Firmicutes | Clostridia | Clostridiales | Mogibacterium_f | HQ400016_g | Mogibacterium_f HQ400016_g |
| Bacteria | Firmicutes | Clostridia | Clostridiales | Lachnospiraceae | FJ681385_g | Lachnospiraceae FJ681385_g |
| Bacteria | Firmicutes | Clostridia | Clostridiales | Ruminococcaceae | AB185816_g | Ruminococcaceae AB185816_g |
| Bacteria | Firmicutes | Clostridia | Clostridiales | Ruminococcaceae | FJ880290_g | Ruminococcaceae FJ880290_g |
| Bacteria | Firmicutes | Clostridia | Clostridiales | Ruminococcaceae | EU259405_g | Ruminococcaceae EU259405_g |
| Bacteria | Firmicutes | Clostridia | Clostridiales | Ruminococcaceae | Eubacterium_g23 | Ruminococcaceae Eubacterium_g23 |
| Bacteria | Firmicutes | Clostridia | Clostridiales | Christensenellaceae | AM277985_g | Christensenellaceae AM277985_g |
| Bacteria | Firmicutes | Clostridia | Clostridiales | Syntrophomonadaceae | Syntrophomonas | Syntrophomonadaceae Syntrophomonas |
| Bacteria | Firmicutes | Clostridia | Clostridiales | Christensenellaceae | EU844999_g | Christensenellaceae EU844999_g |
| Bacteria | Firmicutes | Clostridia | Clostridiales | Ruminococcaceae | AB596889_g | Ruminococcaceae AB596889_g |
| Bacteria | Firmicutes | Clostridia | Clostridiales | Eubacteriaceae | Pseudoramibacter | Eubacteriaceae Pseudoramibacter |
| Bacteria | Firmicutes | Clostridia | Clostridiales | Ruminococcaceae | EF602946_g | Ruminococcaceae EF602946_g |
| Bacteria | Firmicutes | Clostridia | Clostridiales | Lachnospiraceae | EU842423_g | Lachnospiraceae EU842423_g |
| Bacteria | Firmicutes | Clostridia | Clostridiales | Ruminococcaceae | Eubacterium_g8 | Ruminococcaceae Eubacterium_g8 |
| Bacteria | Firmicutes | Clostridia | Clostridiales | Ruminococcaceae | EF404788_g | Ruminococcaceae EF404788_g |
| Bacteria | Firmicutes | Clostridia | Clostridiales | Christensenellaceae | EU844352_g | Christensenellaceae EU844352_g |
| Bacteria | Firmicutes | Clostridia | Clostridiales | Ruminococcaceae | BBZZ_g | Ruminococcaceae BBZZ_g |
| Bacteria | Firmicutes | Clostridia | Clostridiales | Lachnospiraceae | DQ904798_g | Lachnospiraceae DQ904798_g |
| Bacteria | Firmicutes | Clostridia | Clostridiales | Eubacteriaceae | Anaerofustis | Eubacteriaceae Anaerofustis |
| Bacteria | Firmicutes | Clostridia | Clostridiales | Ruminococcaceae | EF559154_g | Ruminococcaceae EF559154_g |
| Bacteria | Firmicutes | Clostridia | Clostridiales | Ruminococcaceae | EU381487_g | Ruminococcaceae EU381487_g |
| Bacteria | Firmicutes | Clostridia | Clostridiales | Ruminococcaceae | FJ951854_g | Ruminococcaceae FJ951854_g |
